# Two Strategies for the Delivery of IPTc in an Area of Seasonal Malaria Transmission in The Gambia: A Randomised Controlled Trial

**DOI:** 10.1371/journal.pmed.1000409

**Published:** 2011-02-01

**Authors:** Kalifa A. Bojang, Francis Akor, Lesong Conteh, Emily Webb, Ousman Bittaye, David J. Conway, Momodou Jasseh, Virginia Wiseman, Paul J. Milligan, Brian Greenwood

**Affiliations:** 1 Medical Research Council Laboratories, Banjul, The Gambia; 2 Institute of Global Health Innovation, Imperial College London, London, United Kingdom; 3 London School of Hygiene & Tropical Medicine, London, United Kingdom; Walter and Eliza Hall Institute of Medical Research, Australia

## Abstract

**Background:**

The Expanded Programme on Immunisation (EPI) provides an effective way of delivering intermittent preventive treatment for malaria (IPT) to infants. However, it is uncertain how IPT can be delivered most effectively to older children. Therefore, we have compared two approaches to the delivery of IPT to Gambian children: distribution by village health workers (VHWs) or through reproductive and child health (RCH) trekking teams. In rural areas, RCH trekking teams provide most of the health care to children under the age of 5 years in the Infant Welfare Clinic, and provide antenatal care for pregnant women.

**Methods and Findings:**

During the 2006 malaria transmission season, the catchment populations of 26 RCH trekking clinics in The Gambia, each with 400–500 children 6 years of age and under, were randomly allocated to receive IPT from an RCH trekking team or from a VHW. Treatment with a single dose of sulfadoxine pyrimethamine (SP) plus three doses of amodiaquine (AQ) were given at monthly intervals during the malaria transmission season. Morbidity from malaria was monitored passively throughout the malaria transmission season in all children, and a random sample of study children from each cluster was examined at the end of the malaria transmission season. The primary study endpoint was the incidence of malaria. Secondary endpoints included coverage of IPTc, mean haemoglobin (Hb) concentration, and the prevalence of asexual malaria parasitaemia at the end of malaria transmission period. Financial and economic costs associated with the two delivery strategies were collected and incremental cost and effects were compared. A nested case-control study was used to estimate efficacy of IPT treatment courses.

Treatment with SP plus AQ was safe and well tolerated. There were 49 cases of malaria with parasitaemia above 5,000/µl in the areas where IPT was delivered through RCH clinics and 21 cases in the areas where IPT was delivered by VHWs, (incidence rates 2.8 and 1.2 per 1,000 child months, respectively, rate difference 1.6 [95% confidence interval (CI) −0.24 to 3.5]). Delivery through VHWs achieved a substantially higher coverage level of three courses of IPT than delivery by RCH trekking teams (74% versus 48%, a difference of 27% [95% CI 16%–38%]). For both methods of delivery, coverage was unrelated to indices of wealth, with similar coverage being achieved in the poorest and wealthiest groups. The prevalence of anaemia was low in both arms of the trial at the end of the transmission season. Efficacy of IPTc against malaria during the month after each treatment course was 87% (95% CI 54%–96%). Delivery of IPTc by VHWs was less costly in both economic and financial terms than delivery through RCH trekking teams, resulting in incremental savings of US$872 and US$1,244 respectively. The annual economic cost of delivering at least the first dose of each course of IPTc was US$3.47 and US$1.63 per child using trekking team and VHWs respectively.

**Conclusions:**

In this setting in The Gambia, delivery of IPTc to children 6 years of age and under by VHWs is more effective and less costly than delivery through RCH trekking clinics.

**Trial Registration:**

ClinicalTrials.gov NCT00376155

*Please see later in the article for the Editors' Summary*

## Abstract 2

Bojang and colleagues report a randomized trial showing that delivery of intermittent preventive treatment for malaria in children by village health workers is more effective than delivery by reproductive and child health trekking clinics.

## Editors' Summary

**Background:**

In sub-Saharan Africa, malaria kills 800,000 people, the majority of whom are children, every year. Intermittent preventive treatment (IPT) of malaria is an effective malaria control strategy. IPT involves administration of antimalarial drugs at defined time intervals to individuals regardless of whether they are known to be infected with malaria to prevent morbidity and mortality from the infection. IPT was initially recommended for pregnant women (IPTp) who are given at least two doses of suphadoxine pyrimethamine (SP) during antenatal visits after the first trimester of pregnancy. IPT is also effective in infants (IPTi) and recently IPTi has been rolled out with the administration of three doses of an antimalarial drug during the expanded program of immunization visits. Clinical studies have also shown that IPT is effective at reducing malaria incidence in children (IPTc) by administering SP alone, or in combination with artesunate (AS) or amodiaquine (AQ,) over three intervals during the peak malarial season.

**Why Was This Study Done?:**

The inclusion of IPTp in antenatal visits and IPTi in the expanded program of immunization has effectively scaled up these interventions to the population level. So far, IPTc has only been administered to children within the confines of clinical trials—there is currently no established system for delivery of IPTc. For the scale-up of IPTc to be successful, there needs to be an appropriate point of entry and the roll out of a delivery system that can be generalized to most settings in sub-Saharan Africa. In order to address this issue, the researchers conducted a randomized trial to compare the effectiveness of IPTc delivery to children up to 6 y of age by village health workers (VHW) or by reproductive and child health (RCH) trekking teams (run by the Ministry of Health) in rural areas of The Gambia.

**What Did the Researchers Do and Find?:**

During the 2006 malaria transmission season, the researchers randomly allocated the catchment populations of 26 RCH clinics, each with 400–500 children 6 y of age and under, to receive IPT from an RCH trekking team or from a VHW. Before the trial started, the researchers, accompanied by the district health team, visited all villages in the study area to explain the purpose and methods of the study and to obtain consent from the elders of all participating villages. Eligible children were treated with a single dose of SP plus three doses of AQ given at monthly intervals during the malaria transmission season. The researchers passively monitored malaria incidence throughout the transmission season and at the end of the malaria season, examined a random sample of 40 children from each cluster to measure their temperature, height, and weight and to take a finger-prick blood sample to measure blood hemoglobin and parasite levels (by microscopy of thick blood smears). The researchers recorded the financial costs associated with each delivery strategy (mostly on the basis of staff pay and the financial incentives given to VHWs).

There were 49 cases of clinical malaria in the areas where IPT was delivered through RCH clinics and 21 cases in the areas where IPT was delivered by VHWs. In addition, VHW delivery of IPTc achieved a higher coverage level of three courses of IPT than delivery by RCH trekking teams (74% versus 48%). The prevalence of anemia was low in both arms at the end of the transmission season. Delivery of IPTc by VHWs was cheaper than delivery through RCH trekking teams, resulting in incremental savings of US$872 and US$1,244, respectively. The annual economic cost of delivering at least the first dose of each course of IPTc using the RCH trekking team was US$3.47 per child and with VHWs was US$1.63 per child.

**What Do These Findings Mean?:**

The results of this study show that in rural areas of The Gambia, delivery of IPTc by VHWs is more effective and less costly than delivery by RCH trekking teams through RCH clinics. Delivering IPTc through community-based VHWs versus monthly visits by the RCH team has several advantages: VHWs are resident in the community, making drug administration easy and flexible (as children were able to receive their medication on any day of the month), and they can remind mothers/guardians to attend for treatment. Therefore, operationally, VHW delivery is less restrictive and more convenient for parents and guardians.

**Additional Information:**

Please access these Web sites via the online version of this summary at http://dx.doi.org/10.1371/journal.pmed.1000409.

- This topic is further discussed in two *PLoS Medicine* research articles by Dicko et al. and Konat et al., and a *PLoS Medicine* Perspective by Beeson
- WHO provides information about The Gambia
- WHO also provides information about the health workforce, including the role of village health workers
- Roll Back Malaria has information about malaria in children, including intervention strategies
- Unicef also provides comprehensive information about malaria in children

## Introduction

Trials conducted in a number of sub-Saharan African countries have shown that antimalarial chemoprophylaxis reduces malaria morbidity and mortality in children and school absenteeism [1]–[5]. However, this approach to malaria control has rarely been implemented widely owing to concerns over its possible effect on the propagation of resistance to antimalarials, the development of natural immunity to malaria, and logistic constraints [6],[7]. To take advantage of the protective effect of chemoprophylaxis whilst reducing the possible adverse effect of chemoprevention on the development of natural immunity to malaria, the concept of intermittent preventive treatment (IPT) with an effective antimalarial has been developed. IPT involves administration of a full treatment dose of an antimalarial drug at specific times, regardless of the presence or absence of malaria parasites. Since treatment is only given intermittently, this intervention is less likely to interfere with the development of natural immunity than sustained chemoprophylaxis.

The IPT strategy was explored first in pregnant women (IPTp) [8]–[10]. The success of IPTp in reducing maternal anaemia and low birth weight generated interest in the use of IPT for the prevention of malaria and malaria-associated anaemia in infants (IPTi) [11]. Following a number of successful trials that showed that IPTi with sulphadoxine pyrimethamine (SP) is safe and reduces the incidence of malaria in the first year of life by about 30% [12], WHO now recommends IPTi as a malaria control strategy in areas where the burden of malaria in infants is high and where resistance to SP is low. However in many countries of the Sahelian and sub-Sahelian regions of Africa where transmission of malaria is highly seasonal, the main burden of malaria morbidity and mortality is not in infants but in older children [13]–[15]. In these countries, there is a need to find new methods for preventing malaria in older children. IPT provides a possible way of doing this.

IPT in children (IPTc) with SP, given alone or in combination with artesunate (AS) or amodiaquine (AQ), reduced the incidence of malaria in children under the age of 5 y in Mali [16] and Senegal [17], countries with a short malaria transmission season, and in an area of Ghana where the malaria transmission season is longer [18]. In recent, large-scale trials conducted in Burkina Faso and Mali, IPTc with SP plus AQ reduced the incidence of both complicated and uncomplicated malaria in children under the age of 5 y who slept under an insecticide-treated bednet (ITN) by approximately 70% [19],[20], indicating that this is a very promising strategy for malaria control in areas where malaria transmission is seasonal. However, in contrast to IPTi and IPTp, which are delivered through established Expanded Programme on Immunisation (EPI) and antenatal clinics, respectively, there is no established system for delivery of IPTc.

In rural areas of The Gambia, there are two possible routes through which IPT might be given to older children: reproductive and child health (RCH) trekking teams and community (village) health workers (VHWs). We have, therefore, undertaken a randomised trial in which we compared the effectiveness of delivery of IPTc to Gambian children 6 y of age and under by VHWs or by RCH trekking teams.

## Materials and Methods

### Study Site and Population

Study participants were recruited from villages and hamlets on the south bank of Upper River Region (URR), The Gambia. The study was conducted between September 2006 and June 2007. In this area, malaria transmission is highly seasonal, occurring mostly during and immediately after the rainy season (July–November) [21]. Usage of ITNs is relatively high with 58.5% of households having at least one ITN in a survey undertaken in 2006 [22]. At the time of this study, first-line treatment for uncomplicated malaria in The Gambia was chloroquine plus SP. In 2001, the PCR-corrected treatment failure rate in symptomatic children at day 28 after the start of treatment with SP and chloroquine was 6% in the central part of the country [23]. Since 2008, first-line treatment has been changed to lumefantrine-artemether (Coartem, Novartis). The study protocol and CONSORT checklist can be found in the supporting information (Text S1 and S2, respectively).

### Overall Study Design

The study was designed as an open-label, cluster randomised trial with the unit of randomisation being the catchment population of RCH trekking teams. There were 33 trekking clinics in the study area on the south bank of URR of which 27 were in rural areas and visited by trekking teams from health centres at Gambisara, Basse, or Fatoto. 26 rural trekking clinics were selected for inclusion in the study (one was excluded to give an equal number in each study arm). The catchment populations of these clinics were grouped into three strata on the basis of the number of children under 5 y of age in each catchment area, and the average distance from the nearest health centre. It was intended that only children under five would be enrolled. However at the time of enrolment about 16% of the children enrolled were more than 5 years. Hence the trial and the analysis were expanded to include children up to and including the age of 6.

Within each stratum, clinic catchment areas were randomised, using permuted blocks using the random number generator in Stata version 10 (Stata Corporation), to receive IPTc delivered either by a VHW or through the RCH trekking team. The randomisation was well balanced with respect to distance from the nearest health centre, population of children up to 6 y of age, proximity to a main road, and quality of the road, factors that might influence access to the intervention and to malaria diagnosis and treatment. For all the villages in the study area, the nearest health centre could be reached in less than 4 h by local transport or was within a few hours walking distance.

### Screening and Enrolment

Following discussions with the National Malaria Control Programme (NMCP) and the URR district level health authorities, members of the study team, accompanied by the district health team, visited all villages in the study area, explained the purpose and methods of the study, and answered questions during meetings open to all villagers. During these meetings, consent was obtained from the elders of all villages in the study area to participate in the trial. Before the start of the study, field workers and investigators carried out a census in each of the 172 villages in the study area that agreed to participate in the trial. A list of all children living in these villages who would be aged between 6 and 72 mo at the time of the first treatment in September 2006 was obtained from this database to ensure that all the children in the right age group were considered for inclusion in the study. Families of prospective study participants were visited at home, where more information was provided about the trial and individual, written informed consent for a child to participate in the trial was obtained from parents or guardians. Exclusion criteria included known allergy to any antimalarial drug or the presence of acute or chronic, clinically significant pulmonary, cardiovascular, hepatic, or renal disease.

A reporter employed by the project was based in each village and asked to keep records of all deaths, birth, immigrations, and emigrations that occurred during the study period. Village reporters were visited fortnightly by project field workers and their records collected and checked. Deaths that occurred at home were investigated with a standardised verbal-autopsy questionnaire and cause of death established whenever possible [24].

### Drug Administration

Drugs were delivered by either members of RCH trekking teams or by VHWs. Study participants were observed for approximately 30 min after dosing and, if vomiting occurred within this period, it was recommended that the child take another dose. RCH trekking teams visited each of the study villages approximately once a month to administer routine EPI vaccines and vitamin A. During the course of the intervention each trekking team in the RCH arm of the trial comprised an average of ten staff members, four of whom were provided by the study team to assist with the intervention. Trekking teams comprised staff with varying levels of training ranging from state enrolled nurses to volunteers. Each member of the team employed by the Ministry of Health received a salary supplement of US$28 per month (US$ 2006) (about 47% of their salary) during the 3 mo of the intervention as an incentive for the extra hours of work that administration of IPTc incurred. Trekking team drivers received a supplement of US$14 per month.

All villages in The Gambia with a population of approximately 400 or more have provision for a VHW who receives limited training from the Ministry of Health on the recognition and treatment of common illnesses including malaria. VHWs are meant to be supported by their community and do not receive any payment from the Ministry of Health. VHWs retain a small stock of essential drugs, including antimalarials, but as these must be purchased from a government pharmacy they frequently run out of stock. Thus, for the period of the study, all VHWs in the study were supplied with antimalarials by the project for the period of the study. VHWs received US$11 per month (US$ 2006) during the period of drug administration and a driver with a supplement of US$14 per month to deliver drugs to the VHWs. VHW supervisors did not receive any additional payment. Before the start of the study, VHWs were trained for 5 d on how to distribute monthly IPTc using drug treatment charts, recognise signs and symptoms of malaria and their relation to severity of illness, and maintain a compliance ledger. Study participants from villages within the IPTc arm of the study but without a resident VHW received IPTc from the nearest village with a VHW.

A system for recording drug administration that could be used by a VHW was devised, which consisted of a treatment card held by the mother or guardian of each child and an IPT register held by the VHW or RCH trekking team. The treatment card and the IPT register were labelled with the child's, mother's, and village's names and compound, and with demographic surveillance and study numbers. A set of three sticky labels were preprinted with the child's identifiers and placed in an envelope that was stapled onto the treatment card. The dosage of trial medication for each study participant was indicated on a largely pictorial treatment card, employed because of the low levels of literacy of some VHWs, using coloured circles and semicircles (a full circle for one tablet and semicircle for half a tablet). To ensure that a child did not receive more doses of IPTc than they should, blue-coloured treatment cards were issued to children in villages where IPTc was to be delivered by VHWs and pink-coloured cards to those allocated to receive medication from the RCH trekking clinic. When a child presented to the VHW or RCH trekking clinic for their monthly treatment, one sticky label was removed from the envelope and attached to the IPTc register, the child's identity checked and trial medication SP (tablets containing 500 mg sulfadoxine/25 mg pyrimethamine) (Microlabs) and AQ (200 mg base tablets) (Microlabs) were given. A single dose of SP was given; children aged 3–11 mo received one half tablet and a whole tablet was given if a child was aged 1–6 y. AQ was given daily for 3 d: one quarter of a tablet if a child was aged 3–11 mo, one half of a tablet if aged 1–2 y, and a whole tablet if aged 3–6 y. SP and the first dose of AQ were given under direct observation by the VHW or member of the trekking team. The mother or carer was given tablets for the second and third dose of AQ in a small plastic bag and was asked to administer these on the following 2 d at home. Children were given their tablets crushed, suspended in water, and administered with a spoon. The solubility and drug content of the SP and AQ tablets was confirmed by analysis with high performance liquid chromatography undertaken at the London School of Hygiene & Tropical Medicine.

### Surveillance for Adverse Events

To determine the prevalence of adverse events following drug administration, 15 children in each cluster selected from the participant database by simple random sampling were visited at home within a week of the start of each round of drug administration by a trained field worker who completed an adverse event questionnaire. Parents and guardians of study children were encouraged to take their child to the study nurse based in the village or to the Medical Research Council (MRC) clinic at Basse if the child became unwell between visits. Any adverse reaction was graded by trained field workers as mild (grade 1) if it was easily tolerated, moderate (grade 2) if it interfered with normal activity, and severe (grade 3) if it prevented normal activity and required treatment. In addition, records were kept of all deaths and hospital admissions among study children during the period of the trial. A serious adverse experience was defined as any event that was fatal, life threatening, disabling or incapacitating or resulted in hospitalisation, prolonged a hospital stay, or was associated with overdose (either accidental or intentional). A decision was made by the study physician as to whether a serious adverse event was likely to be drug related.

### Morbidity Surveillance during the Rainy Season

Passive surveillance for malaria was maintained throughout the malaria transmission season (September to December). In both arms of the trial, study children had 24-h access to medical treatment provided by VHWs based in key villages or by study nurses based in the health centres within the study area. In both arms of the study, VHWs were asked to refer study participants who presented to them to the nearest health facility for evaluation. Each time a study patient presented to a health facility, axillary temperature was recorded using a digital thermometer and haemoglobin (Hb) concentration was measured using a portable haemoglobinometer (HemoCue AB). The Core Malaria Pf test (CORE Diagnostics), based on detection of circulating *Plasmodium falciparum* histidine-rich protein 2 antigen, which is sensitive and specific for detection of *P. falciparum* in whole blood samples, was used to diagnose malaria if fever (axillary temperature ≥37.5°C) or a history of fever within the previous 48 h was present. In such cases, a thick blood smear was also collected for subsequent confirmation of the diagnosis and estimation of parasite density. Study participants with documented fever or history of recent fever and malaria parasitaemia were treated with Coartem. Treatment of study participants seen at the health centres for other conditions was carried out in accordance with national guidelines.

A cross-sectional survey was undertaken in all 26 clusters at the end of the malaria transmission season. Children in each cluster were selected for review by simple random sampling from the list of study participants. The families of children selected for review were visited at home, and follow-up visits made to find children who were not present at the first visit. The first 40 children to be located in each cluster were included in the survey. Children included in the survey were examined, their axillary temperature measured, and their height and weight recorded. A finger-prick blood sample was taken for preparation of thick smears and measurement of Hb concentration. The dates of IPT treatments received were recorded from the child's identification (ID) card, and the mother or career was asked about reasons for missed treatments, about the acceptability of the most recent round of treatment, and about compliance with the unsupervised doses. The families of the same children were visited 2–3 wk later and asked questions about household assets, level of education and sources of income of the child's primary care giver, the care giver's perception of the relative wealth of the household, bednet use by the study child, and asked to inspect the place where the child usually slept and to inspect the condition of their net if a net was being used.

### Case-Control Study

In order to estimate efficacy of IPTc, a nested case-control approach was used. Cases were children who presented to one of the study health centres with an axillary temperature ≥37.5°C or a history of fever in the previous 48 h and who had malaria parasitaemia with a parasite density ≥5,000/µl on initial reading. Controls (initially two per case and subsequently one per case) were selected from among children who presented to the same health centre with a febrile illness in the same week that the case was detected, who had a negative blood film, and whose village of residence was in the same delivery arm (VHW or RCH) as the case child. Cases and controls were visited at home to inspect the place where the child slept, to ask the child's mother or carer about bednet use at the time that the case child became sick, and about household assets. Dates of IPT treatment courses were obtained from the child's ID card and cross-checked against study registers.

### Laboratory Methods

Thick smears were stained with Giemsa stain and 200 high power fields (HPFs) were examined before a smear was declared negative. Parasite density was expressed per microlitre with the assumption that one parasite per HPF equals a density of 500 parasites per microlitre [25]. All slides were read independently by two laboratory technicians. If there was disagreement between their readings on parasite positivity or if the difference in the log_10_ densities recorded was more than 1.5, slides were read by a third technician. Agreement was reached among the three technicians after the slides had been rechecked. Discrepancies occurred mainly in smears with very low parasite densities. Hb concentration was measured during morbidity surveillance and at the survey at the end of the malaria transmission season using a portable haemoglobinometer (Hemocue AB).

### Collection of Cost Data

A standard ingredients approach was used to cost the value of each unit of input needed to deliver IPTc and to treat inpatient or outpatient malaria episodes [26],[27]. We report both financial costs (additional government expenditure) and economic costs (the costs of all resources needed to deliver IPTc regardless of funding sources). For both the VHW and RCH trekking team, IPTc delivery costs were broken down by categories including IPTc drugs, non-IPTc drugs, drug dispensing, drug distribution, supervision, training, and supplies. In addition, household costs of receiving IPTc were based on a questionnaire completed by a sub sample of 15 participants attending each of the 26 maternal and child health clinic sites (*n* = 390). Fieldworkers were asked to stagger their interviews over the course of the day to ensure that care givers accessing IPTc at various times could be included.

To reflect potential health system savings associated with fewer malaria episodes, economic costs of treating malaria were based on detailed retrospective cost data from health facilities across primary, secondary, and tertiary level health facilities in the study area between 2006 and 2007. Resource use associated with personnel, materials and supplies, equipment, transport, utilities, and buildings were recorded. Costs were identified using information found in patient folders, facility stock records, activity data collected by the district medical team, discussions with health facility personnel (both medical and administrative), and components of the study budget. Potential savings to households (direct and indirect), such as reduced expenditure on transport and other out-of-pocket expenses and potential savings in time lost from seeking treatment were collected from the families of all study children who were admitted to a hospital or health centre and from the families of a subsample of 100 children treated as outpatients. Costs are reported in 2008 US$.

In order to calculate the incremental cost-effectiveness ratios, the costs of delivering IPTc via RCH trekking teams were compared with the costs of delivery by VHWs and this difference in costs was then compared to the following additional effects: (i) number of malaria episodes averted; (ii) number of children receiving the first dose of all three treatments; and (iii) number of children who received the first dose of at least one treatment. For ethical reasons, no placebo group was included in the current study so it was not possible to compare cost-effectiveness of these two strategies to current practice. Net cost effectiveness was calculated for each delivery system by subtracting resources saved from the total programme compared to the relevant outcome measure.

### Data Management

Data from participants were recorded on forms and were checked by field supervisors, the study physician, and the data manager for consistency and accuracy. All data were entered into an SQL database using MS Access front-end software. The accuracy of data input was checked and validated using customized validation programmes. In addition, source data verification was done by the data manager. The cleaned data were converted to Stata version 10 file (Stata Corporation) prior to analysis.

### Statistical Analysis

The primary study endpoint was the incidence of malaria (documented fever [axillary temp ≥37.5°C] or a history of fever within the previous 48 h accompanied by asexual malaria parasitaemia at a density of ≥5,000 parasites/µl) observed during the study period. Secondary endpoints were the incidence of a febrile illness with parasitaemia at any density, coverage of three IPTc treatment courses, the proportion of children who received no IPTc, the incidence of adverse events reported after IPTc administration, the mean Hb concentration, and the prevalence of asexual parasitaemia in the cross-sectional survey undertaken at the end of the malaria transmission season. The study was designed to determine whether delivery of IPTc through RCH trekking clinics would be at least as effective in preventing malaria as delivery by VHWs, and the sample size was calculated on this basis. On the basis of previous studies carried out in The Gambia, we assumed that 75% coverage with three IPT treatment courses would be achieved in both arms, that the efficacy of IPT would be 90%, and that the incidence rate of malaria by passive case detection would be 0.2 per child per transmission season without IPT. The predicted cumulative incidence of malaria would therefore be 6.5%, and we assumed that malaria incidence in each cluster would range from 0% to 20%. With 26 clusters and a cluster size of 500 children per cluster, the study was designed to have 90% power to show (using a 95% confidence interval [CI]) that the difference in cumulative incidence between the delivery methods was not more than 3.5%. Similar calculations were done for coverage of IPTc, using a noninferiority margin of 10% to 15%, and for mean Hb at the end of the transmission season, using a margin of 0.5 g/dl. An analysis plan was agreed before the data were analysed.

The primary analysis included all children in the correct age range who were present in the study area at the initial census and were issued with a malaria card, regardless of the number of treatments received.

Characteristics of all children enrolled in the study were tabulated by study arm, overall estimates (not adjusting for clustering) are presented. For the primary outcome of malaria incidence during the study period, analysis was by intention to treat. Time at risk was calculated from September 11 to December 8 (the surveillance period) or until date of death or emigration. All episodes of malaria were included in the calculations; only two children had two episodes of malaria. The incidence of malaria in each study arm was calculated as the total number of cases divided by the total time at risk. The point estimate of the rate difference between the two arms was the difference between these rates and a 95% CI adjusting for clustering, and strata was calculated from the variances of the stratified ratio estimates [28]. An adjusted rate difference was calculated using a two-stage approach [29]. Both individual and village-level covariates (child's age, sex and ethnicity, and village population and distance to nearest health centre) were included in a Poisson regression model, the adjusted rate difference and 95% CI were then estimated from the model residuals. Some covariates likely to be prognostic for malaria risk, for example net use, were only measured after the intervention period and on a subset of children and were therefore not adjusted for.

For the analysis of the efficacy of IPTc against clinical malaria using the case-control approach, since some cases had parasitaemias <5,000/µl on definitive reading, two case definitions of malaria were used: a febrile illness accompanied by malaria parasitaemia of any density or by malaria parasitaemia at a density ≥5,000 per µl. The efficacy of an IPTc course given within the previous 28 d in preventing malaria was estimated as 100× (1−1/OR) in which OR is the odds ratio estimated by conditional logistic regression relating case/control status to IPTc. Bednet usage (defined as usually slept under an intact or treated net, and the net could be tucked under the mattress), age, ethnicity, self-reported wealth, and distance to the nearest health centre were considered as potential confounders. In a secondary analysis, interaction with bednet usage was assessed using a likelihood ratio test, to determine whether there was any difference in the benefit of IPTc in children using bednets compared to children not using nets. Interactions with delivery method and socioeconomic variables were also assessed.

Coverage with IPTc was evaluated in the cross-sectional sample survey conducted at the end of the study. The proportions of children who had received each monthly treatment course, those who received three treatment courses, and those who received no treatment were tabulated. Differences in coverage estimates between trial arms were estimated by calculating the arithmetic mean of coverage proportions in each cluster, and conducting a two-way analysis of variance, allowing for stratification.

To determine whether one delivery method was better at reaching particular subgroups of the population, the coverage indicators were tabulated, stratified by wealth, and by age of each child. Wealth quintiles were calculated as quintiles of the scores on the first component from principal components analysis of household asset ratings (based on household ownership of ten items), as recorded in the cross-sectional survey.

Numbers of adverse events were tabulated by study arm, and risk differences with 95% CIs were calculated adjusting for clustering and strata using analysis of variance on the arithmetic cluster means, as described above. Outcomes collected at the December cross-sectional survey were also analysed using the analysis of variance method described above.

### Ethical Review

The study was approved by the London School of Hygiene & Tropical Medicine ethics committee and by the joint MRC/Gambia Government ethics committee. The conduct of the trial was guided by a Data Safety and Monitoring Board. The trial was registered on the NCHS clinical trials database (number NCT00376155).

## Results

### Baseline Comparability

**Figure 1:**
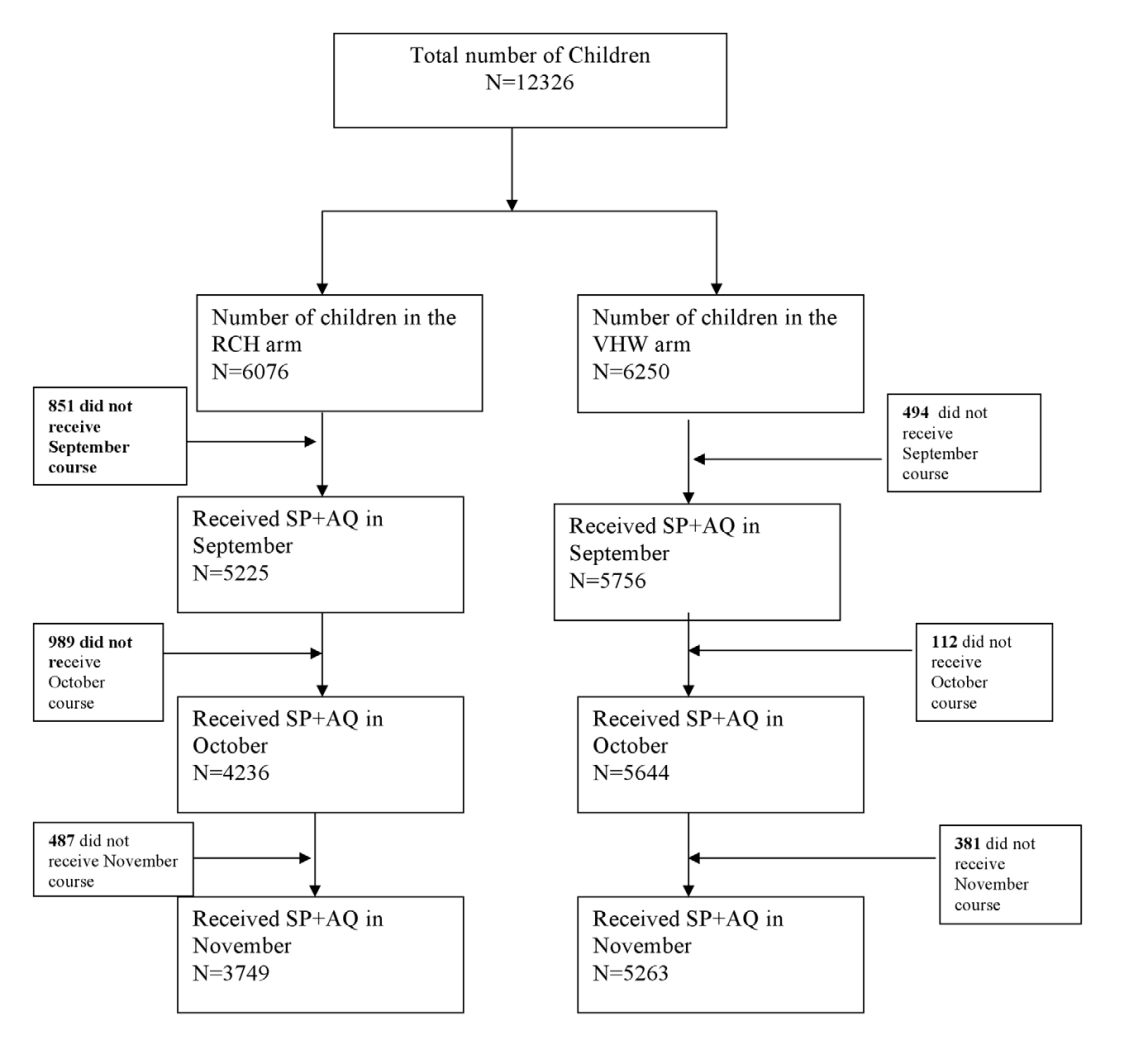
Trial profile.

12,326 children aged 6 mo to 6 y were enrolled into the study, of whom 6,076 were in the RCH arm and 6,250 in the VHW arm (Figure 1). Baseline characteristics of children in each arm of the study are shown in Table 1. The number and age distribution of children in the two study groups were similar. Observations made during the cross-sectional survey undertaken at the end of the malaria transmission season suggested that a slightly higher proportion of children in the VHW arm slept under a bed net compared to those in the RCH arm and that more children in the VWH group were fully vaccinated compared to those in the RCH arm.

**Table 1:** Baseline characteristics of the children in the two study groups.

Baseline Characteristics	RCH Delivery	VHW Delivery
***Total population***	40,115	38,681
Number of children ≤6 y	6,076	6,250
***Age (mo)***		
<12	480 (7.9%)	470 (7.5%)
12–23.9	1,243 (20.5%)	1,281 (20.5%)
24–35.9	1,161 (19.1%)	1,181 (18.9%)
36–47.9	1,149 (18.9%)	1,241 (19.9%)
48–59.9	1,021 (16.8%)	1,072 (17.2%)
≥60	1,022 (16.8%)	1,005 (16.1%)
***Gender (48 missing)***		
Male	3,071 (50.6%)	3,165 (50.9%)
***Ethnic group (65 missing)***		
Fula	1,986 (32.8%)	1,985 (32.0%)
Sarahule	2,671 (44.1%)	2,914 (46.9%)
Mandingo	1,288 (21.3%)	1,222 (19.7%)
Others	107 (1.8%)	88 (1.4%)
***n Clusters***	13	13
***n Villages***	89	80
***Village population size (mean, range)***	446 (13–4,758)	478 (6–4,251)
***Total n VHWs***	13	18
***Mean (range) distance to nearest health centre (km)***	14.2 (3–34)	13.9 (4–30)

### Coverage Achieved with the Two Delivery Methods

On the basis of data collected during the cross-sectional survey undertaken at the end of the surveillance period, 92% and 86% of participants issued with a malaria card at time of the census reported for enrolment and received the first IPTc treatment in the VHW and RCH arms, respectively. More children in the VHW arm received three treatment courses compared to the RCH arm (74% versus 48%, a risk difference of 27% [95% CI 16%–38%]). Coverage was higher in the VHW arm than in the RCH arm for each monthly treatment round (Figure 1). In the RCH arm, coverage was initially high but decreased progressively (Table 2). In the VHW arm, coverage was slightly lower in November than in September and October. During the November treatment round, 98% of children were reported to have received both additional AQ doses at home, and 5% of those who received treatment were reported to have vomited the drug. These figures were similar for each delivery method. For both delivery methods, coverage with the three treatments was similar in each socioeconomic group defined by wealth quintiles derived from household assets. In the VHW arm, coverage of three courses of IPT was 76% in the wealthiest quintile and 73% in the poorest quintile; in the RCH arm coverage of three courses was 40% in the wealthiest quintile and 48% in the poorest quintile.

**Table 2:** Coverage of monthly IPT treatments achieved with delivery of IPTc by RCH trekking teams or by VHWs.

Monthly IPT Treatments	RCH (*n* = 511)	VHW (*n* = 550)	Difference (95% CI)^a^
Received September treatment (7 missing)	437 (86.0%)	503 (92.1%)	6.6% (−0.3% to 13.6%)
Received October treatment (7 missing)	357 (70.3%)	493 (90.3%)	20.3% (13.1%–27.6%)
Received November treatment (6 missing)	314 (61.7%)	460 (84.2%)	22.1% (12.3%–32.0%)
Received 3 treatments (9 missing)	242 (47.8%)	406 (74.4%)	26.7% (15.6%–37.9%)
Received 0 treatments (9 missing)	36 (7.1%)	11 (2.0%)	−5.2% (−9.1% to −1.3%)

a Adjusted for clustering and strata.

### Safety and Tolerability of IPTc with SP plus AQ

18 study children died during the surveillance period, nine in each study arm. In the VHW group, five children died of severe pneumonia, two children died of acute gastroenteritis, one child died of cerebral malaria, and one from the complications of severe malnutrition. In the RCH arm, three children died of severe pneumonia, two died of malnutrition, two of acute gastroenteritis, and two of severe malaria. 68 study children were admitted to one of the health facilities in the study area during the follow-up period; 30 and 36 in the RCH and VHW groups, respectively. The main causes of admissions were malaria and pneumonia. 24 children were diagnosed with severe malaria, 14 in the RCH group and 10 in the VHW group. 30 children were diagnosed with pneumonia, 13 and 17 in the RCH and VHW groups, respectively. None of the deaths or hospital admissions was considered to be related to drug administration. There were no deaths or hospital admissions associated with severe skin lesions and no deaths suggestive of a blood dyscrasia.

Field workers visited 838 study participants within the first week after the start of IPT treatment to document the incidence of adverse reactions. The treatment regimen was generally well tolerated. The most frequently solicited adverse events reported after treatment were fever, vomiting, diarrhoea, and abdominal pain, which were reported by 24%, 12%, 13%, and 5% of children, respectively. Seven children reported a rash but in no child was this severe.

### Incidence and Prevalence of Malaria in the Two Study Populations

VHWs in the study area presumptively treated 288 study participants for malaria (160 in VHW group, 128 in RCH group); these were mainly those whose parents/guardians were unable or were unwilling to travel to health centre. There were 49 cases of malaria with parasitaemia >5,000/µl in the communities where IPTc was delivered through RCH clinics and 21 cases in the communities where IPT was delivered by VHWs, (incidence rates 2.8 and 1.2 per 1,000 child months, rate difference 1.6 [95% CI −0.24 to 3.5]), and 144 cases with any parasitaemia in the RCH areas compared to 81 in the VHW areas (rate difference 3.7 [95% C1 −0.7 to 8.1) (Table 3).

**Table 3:** Incidence of clinical episodes of malaria in the two study groups.

Episodes	RCH			VHW			Rate Difference (95% CI)^a^	Adjusted Rate Difference (95% CI)^b^
	Total Events	Child Months at Risk	Incidence Rate/1,000 Child Months	Total Events	Child Months at Risk	Incidence Rate/1,000 Child Months		
Episodes with >5,000 parasites per µl	49	17,561	2.79	21	18,055	1.16	−1.63 (−3.49 to 0.24)	−1.49 (−3.17 to 0.18)
Episodes with any asexual stage parasitaemia	144	17,561	8.20	81	18,055	4.49	−3.71 (−8.12 to 0.70)	−3.89 (−7.48 to −0.29)

a Adjusted for clustering and strata.

b Adjusted for child's age, sex, and ethnicity; village population and distance to nearest health centre; clustering and strata.

1,096 children were seen during the December 2006 survey at the end of the malaria transmission season (Table 4). Very few children were parasitaemic; the proportion of children who had positive blood slides was similar in both groups (3.3% in the RCH group and 2.3% in the VHW group) and fewer children had malaria parasitaemia of ≥5,000 parasites/µl (0.6% in the RCH group and 1.1% in the VHW group). Splenomegaly was also very infrequent, being present in 1.8% and 1.3% of children in the RCH and VHW groups, respectively. Mean Hb concentration was similar in each group (10.3 g/dl RCH group versus 10.4 g/dl VHW group). The proportions of children with moderate anaemia (Hb <7 g/dl) were 6.1% and 4.8% among children in RCH and VHW groups, respectively.

**Table 4:** Results of end of rainy season cross-sectional survey.

Results	RCH (*n* = 525)	VHW (*n* = 571)	Difference^a^ (95% CI)
*P. falciparum* parasitaemia ≥5,000 parasites/µl (23 missing)	4 (0.8%)	6 (1.1%)	0.3% (−1.2% to 1.8%)
Any *P. falciparum* parasitaemia (23 missing)	17 (3.3%)	13 (2.3%)	−1.1% (−5.1% to 3.0%)
Geometric mean density (95% CI) of positive slides	337 (69–1,648)	478 (55–4,190)	
Splenomegaly (60 missing)	9 (1.7%)	7 (1.3%)	−0.8% (−2.9% to 1.4%)
Mean Hb concentration (SD)	10.2 (2.0)	10.4 (2.0)	0.16 (−0.22 to 0.54)
Anaemia (Hb <11 gm/dl; 9 missing)	314 (59.9%)	325 (57.7%)	−1.8% (−9.5% to 5.9%)
Moderate anaemia (Hb <7 gm/dl; 9 missing)	32 (6.1%)	27 (4.8%)	−1.2% (−4.5% to 2.2%)
Height-for-age *z*-score < −2 SD	93 (21.5%)	112 (23.4%)	3.7% (−2.6% to 10.0%)
Weight-for-age *z*-score < −2 SD	152 (30.5%)	156 (31.3%)	2.5% (−5.0% to 10.2%)
Weight-for-height *z*-score < −2 SD	98 (22.9%)	93 (19.4%)	−0.4% (−7.9% to 7.1%)
Usually sleeps under a bednet (32 missing)	314 (64%)	379 (71%)	
Usually sleep under an intact or treated net^b^ (32 missing)	252 (51%)	343 (64%)	
Children aged ≥12 mo who are fully vaccinated (97 missing)	289 (69%)	371 (79%)	

a Adjusted for clustering.

b Strata treated in the last 12 mo or intact, and can be tucked under a mattress.

SD, standard deviation.

### Efficacy of IPTc in Preventing Clinical Episodes of Malaria

81 cases of malaria and 89 controls were recruited into the case-control study (73 cases had one control and eight cases had two controls). 56 case-control sets came from villages in the RCH study area and 25 from villages in the VHW study area. 18 cases had parasitaemia <5,000/µl on definitive reading of a blood film. 20 (32%) of the 63 cases with parasitaemia ≥5,000/µl had received an IPT course in the 28 d prior to the date the case was detected, compared to 64% (45/70) among controls. All 65 cases and controls who received an IPT course in the 28 d prior to case detection reported full compliance with the additional home-administered AQ doses. The crude estimate of protective efficacy was 82%, and after adjustment for potential confounding effects of bednet use, age, ethnicity, self-reported wealth, and distance to nearest health centre, the efficacy of any IPTc treatment received within the previous 28 d against a febrile illness accompanied by malaria parasitaemia ≥5,000/µl was 87% (95% CI 54%–96%). When cases with parasitaemia <5,000/µl were included, adjusted efficacy was 80% (95% CI 42%–93%). There was no evidence of an interaction between the efficacy of IPTc and bednet use.

### Cost and Cost Effectiveness of IPTc Delivery

Table 5 shows the total financial and economic costs of delivering IPTc via either the RCH trekking teams or through VHWs. The unit costs of delivering IPTc are presented in Table 6. The financial costs are low using either delivery strategy because certain costs associated with IPTc drug delivery and supervision did not necessitate additional government expenditure as these activities were already provided routinely. The difference in drug costs reflects the difference in adherence, but even with lower adherence and therefore lower drug costs, IPTc delivery was more costly overall using RCH trekking teams than when delivered by VHWs. The highest percentage of the total costs associated with delivery by the RCH trekking teams was the costs of drug delivery during the EPI clinics. This difference reflects the higher salary costs (a senior enrolled nurse is paid a salary of approximately US$60 monthly) and the US$28 monthly incentive required to pay trained health care professionals to deliver IPTc. This cost can be compared to the US$11 per month “incentive” given to the VHW for administering IPTc. The highest financial cost component of the VHW strategy was the delivery of non-IPTc drugs. Training costs were similar for both the RCH and the VHW delivery strategies. A total of 20 health care professionals needed to be trained for delivery through the RCH trekking teams compared to the 33 VHWs who needed to be trained to achieve delivery in the community. From a household cost perspective, none of the 390 care givers who were interviewed reported direct costs associated with receiving IPTc and only one reported a loss in actual earnings while accessing IPTc.

**Table 5:** Total costs and cost effectiveness by delivery strategy (US$ 2008).

Categories	Provider Costs Via RCH Trekking Teams				Provider Costs Via VHWs			
	Financial Costs^a^		Economic Costs		Financial Costs		Economic Costs	
	US$	Percent	US$	Percent	US$	Percent	US$	Percent
**Costs**								
Cost of IPTc Drugs	835	17	835	14	1,050	28	1,050	21
Cost of non-IPTc Drugs	887	18	887	15	1,144	30	1,144	22
Drug dispensing	2,072	41	2,072	35	676	18	1,184	23
Drug delivery								
*- Personnel*	—	—	1	0	—	—	64	1
*- Transport*	—	—	262	4	—	—	152	3
Supervision								
*- Personnel*	276	5	429	7	138	4	160	3
*- Transport*	—	—	465	8	—	—	454	9
Training	517	10	559	9	511	13	608	12
Supplies	454	9	454	8	279	7	279	5
**Gross intervention cost:**	**5,042**	**100**	**5,966**	**100**	**3,798**	**100**	**5,093**	**100**
***Incremental saving*** b								
Intervention savings					1,244		872	
Intervention and provider treatment savings					1,443		1,070	
Intervention, provider, and household treatment savings					1,521		1,148	
***ICERs*** c								
Saving per malaria episodes averted					1,9.76		13.84	
Saving per fully adherent child					0.87		0.61	
Saving per child who received at least one dose					4.96		3.48	

a Financial costs reflect the additional resources required to deliver IPTc in terms of the actual expenditures incurred. Economic costs capture the opportunity cost of all resources used to provide IPTc, whether or not they incur a financial cost.

b Incremental estimates reflect the cost savings of VHW over RCH trekking teams and compare these to the additional effects.

c Incremental cost effectiveness ratios (ICERs) are calculated on the basis of intervention costs only.

**Table 6:** Unit costs by delivery strategy (US$ 2008).

Cost Categories	Unit IPTc Provider Costs Via RCH Trekking Teams								Unit IPTc Provider Costs Via VHW							
	Per Child Receiving First Dose of All Three Treatments^a^				Per Child Receiving First Dose of at Least One Treatment				Per Child Receiving First Dose of All Three Treatment				Per Child Receiving First Dose of At Least One Treatment			
	Financial Costs		Economic Costs		Financial Costs		Economic Costs		Financial Costs		Economic Costs		Financial Costs		Economic Costs	
	US$	Percent	US$	Percent	US$	Percent	US$	Percent	US$	Percent	US$	Percent	US$	Percent	US$	Percent
Cost of IPTc Drugs	0.67	23	0.67	19	0.21	23	0.21	19	0.39	32	0.39	24	0.21	32	0.21	24
Cost of non-IPTc drugs	0.49	16	0.49	14	0.15	16	0.15	14	0.35	28	0.35	22	0.19	28	0.19	22
Drug dispensing	1.12	38	1.12	33	0.35	38	0.35	33	0.21	17	0.36	22	0.11	17	0.19	22
Drug delivery																
*- Personnel*			0.00	0			0.00	0			0.02	1			0.01	1
*- Transport*			0.14	4			0.04	4			0.04	3			0.02	3
Supervision																
*- Personnel*	0.15	5	0.23	7	0.04	5	0.07	7	0.04	3	0.05	3	0.02	3	0.02	3
*- Transport*			0.25	7			0.07	7			0.14	9			0.07	9
Training	0.29	10	0.31	9	0.08	10	0.10	9	0.16	13	0.19	11	0.08	13	0.10	11
Supplies	0.24	8	0.24	7	0.07	8	0.07	7	0.08	7	0.08	5	0.04	7	0.04	5
**Total cost**	**2.97**	**100**	**3.47**	**100**	**0.93**	**100**	**1.08**	**100**	**1.23**	**100**	**1.63**	**100**	**0.66**	**100**	**0.87**	**100**

a First dose was under observation.

In terms of cost-effectiveness, this incremental analysis shows that the VHW strategy is both more effective and less costly that the RCH approach. Comparing VHWs against the RCH trekking team there are three positive incremental effects: a reduction in the number of malaria episodes averted by 63, an improvement in the number of fully adherent children by 1,429, and an improvement in the number of children who received at least one treatment by 251. The VHW strategy is also less costly in both economic and financial terms than the RCH approach, resulting in incremental saving of US$872 and US$1,244, respectively, on the basis of intervention costs alone. When we include both intervention costs (the costs of delivering IPTc) and health system savings (the costs averted by fewer in- and out-patient malaria episodes) savings increase to US$1,070 and US$1,443 in economic and financial terms, respectively. Finally, when household savings due to the reduction in malaria treatment costs are also added, by selecting the VHW approach over the RCH approach, the incremental savings increase further to US$1,148 and US$1,521 in economic and financial terms, respectively.

## Discussion

The efficacy results of this trial are consistent with findings from previous studies of IPTc carried out in other West African countries, which have shown significant levels of protection against clinical attacks of malaria during the malaria transmission season [16]–[20]. It was not considered appropriate to include a placebo group in this study because of the clear evidence from previous studies that IPTc is beneficial. However, using a case-control approach, the intervention appeared to be highly efficacious in preventing clinical episodes of malaria with a protective efficacy for an IPT course given within the previous month of over 80%, a figure comparable to that observed in a randomised controlled trial undertaken in neighbouring Senegal [17]. The prevalence of parasitaemia at the end of the malaria transmission season, when this is normally at its highest, was very low in both sets of villages (<3%) and much lower than the rate recorded in the study area in previous years [30], suggesting that in both study arms IPTc had been highly efficacious. However, these low incidence and prevalence figures must be set in the context of an overall, recent decline in the incidence of malaria in The Gambia associated with a high use of ITNs and introduction of more effective treatment [15].

In The Gambia delivery of IPTc by VHWs proved to be more effective than delivery by RCH trekking teams as judged by the level of coverage achieved. This finding is in agreement with the findings of a small-scale study that investigated different options for the delivery of IPTc in Ghana. This study also found that the proportion of children who received at least the first dose of three or more courses of IPTc was slightly higher in the community-based arm compared to delivery by health workers at health centres or at EPI outreach clinics (facility based) [31]. In keeping with the finding that more effective coverage was achieved by VHWs than by the trekking teams, the incidence of malaria, as determined by passive case detection, was less in villages where IPTc was delivered by VHWs than in those where RCH trekking teams were used.

Delivering IPTc through community-based VHWs has several advantages. VHWs are resident in the community, making drug administration easy, and they can remind mothers/guardians if they do not come for treatment. Children were able to receive their medication on any day of the month. In contrast, children in the RCH arm were able to receive their treatment only once a month during the monthly visit of the trekking team, which may explain the lower coverage observed in the RCH arm of the study in October and November. Thus, operationally, VHW delivery is less restrictive and more convenient for parents and guardians. A previous study carried out in The Gambia showed that that VHWs were able to achieve a high level of coverage of antimalarial chemoprophylaxis among children and that in about 50% of the study villages, an acceptable level of compliance was sustained for a period of 5 y, despite minimal outside supervision and support for the programme [32]. Delivering IPTc through the community-based VHWs proved to be the most effective approach in The Gambia; however, this may not be the case in other areas. Thus, there is a need to determine the optimum delivery method in other settings.

A potential weakness of the trial was that it was not a placebo controlled. It was not considered appropriate to include a placebo group in this study because of the clear evidence from previous studies in neighbouring Senegal and elsewhere that IPTc is beneficial. Efficacy of the intervention was estimated using a nested case-control study and, although this is not as rigorous as a randomised trial, the estimates of efficacy were similar to those obtained in previous trials. A further possible limitation is that, although care was taken to ensure balanced randomisation, we did not have prior data on malaria incidence in the study villages, and an imbalance cannot be ruled out. However, the difference in incidence of malaria between the study arms is consistent with the difference in coverage of IPT and the observed efficacy of IPT doses, and so these may not have been important sources of bias. It has to be noted, however, that the CI for the difference in malaria incidence is wide (a general decline in malaria transmission meant there were fewer events than we had anticipated when planning sample size). A further potential source of bias in this study is that VHWs were involved in IPTc delivery and this may have influenced their treatment or referral of suspected malaria patients.

No serious adverse event attributable to study medication was observed following administration of about 30,000 courses of SP and AQ, adding to the increasing body of evidence that this drug combination is safe when used for IPTc in African children. No serious skin lesions were observed and there were no clinical cases suggestive of a blood dyscrasia, although, as white cell counts were not done routinely, the possibility that a blood dyscrasia contributed to one of the fatal infections that occurred among study children cannot be completely excluded. The overall mortality rate among the children in the study was approximately 1.5 per 1,000 during the 4-mo period of observation, which included, the period when most childhood deaths occur in The Gambia, a figure well below that expected on the basis of previous studies conducted in URR [14]. The most frequent minor adverse events reported were fever, diarrhoea, vomiting, and malaise. Reports of vomiting probably included some children who spat out the medicine in reaction to the unpleasant taste of AQ. Since there was no control group, it was not possible to assess the relationship of the adverse events observed in this trial to study drugs and many apparent side effects were likely to have been due to associated infections, which are common during the rainy season. Development of a liquid paediatric formulation would facilitate administration of IPT and might reduce minor adverse events and increase compliance.

We selected SP plus AQ as the combination to be used for IPTc in this study because a trial undertaken in Senegal, which compared four different treatment regimens, showed that the best results were obtained with this regimen [33]. However, because of increasing levels of resistance to SP and the unpalatability of AQ, there is a need to find alternative drugs for IPTc. A recent trial carried out in Senegal showed that combinations containing piperaquine were safe and equally effective in preventing malaria [34] and similar observations have been made in The Gambia [35].

At between US$3.47 (using trekking teams) and US$1.63 (using VHWs), the annual economic cost of delivering at least the first dose of each treatment of IPTc under trial conditions are comparable to the range of the costs associated with delivering other malaria interventions. For example, the costs per year of protection in US$ 2008 are reported to be US$1.46–US$4.00 for ITNs [36], US$3.62–US$6.06 for indoor residual spraying (IRS) [37], US$0.75 for IPT in infants using SP [38] and US$2.02 for IPT in school children [39].

This study has shown the influence of scale on delivery of IPTc. A smaller study of IPTc delivery gave unit costs of village-based delivery more than three times higher than those presented here [40], even after taking into account differences in costs of IPTc drugs and of treating cases of malaria. In the present much larger study, including over 12,000 children, certain fixed costs such as incentives to VHWs and facility-based staff are divided by a much larger number of children. Semi-fixed costs such as training, drug delivery, and supervision also benefit from economies of scale in The Gambia.

There are many potential ways in which VHWs can contribute to the health of the communities in which they are based. These ways include treatment of common infections, recognition and referral of seriously ill patients, provision of advice on hygiene and nutrition, and encouraging women to attend antenatal clinics and to immunize their children. Community volunteers are also being used increasingly as a route of delivery for mass treatment campaigns against infections such as onchcocerciasis, intestinal helminth infections, shistosomiasis, filariasis, and trachoma. In the case of malaria, VHWs can provide treatment for clinical attacks of malaria (home management of malaria) (HMM) either presumptively or following the use of an RDT. They can encourage the use of ITNs and, as we have shown in this study, effectively administer IPTc. In countries, such as The Gambia, where malaria transmission is seasonal, this activity in required for only a few months of the year. However, although the incidence of malaria in The Gambia, and countries with a similar epidemiology, is concentrated within just a few months of the year, occasional cases do occur at other times of the year and require treatment. Thus, a logical approach is to ask VHWs to combine the role of HMM throughout the year with the administration of IPTc during the high transmission season. This approach has recently been explored in The Gambia [41] and shown to be effective and evaluated in Ghana where the malaria transmission season extends over a longer period of the year [31].

Sustainability is a major concern for all interventions, especially those that depend upon volunteers. Given the renewed focus on VHWs and their ever-expanding repertoire of services [42], a system must be identified to ensure that they are adequately trained and supported and not given more tasks than they can cope with [43]. The issue of training frequency and supervision intensity, both for IPTc specifically, and in combination with the other VHW tasks needs further investigation, as it will have implications on both health and cost outcomes. As a component of the trial, VHWs were supplied with antimalarials, oral rehydration solution (ORS), and paracetamol syrup, thus strengthening their role in the community in general. How to guarantee the routine supply of these medicines outside trial settings, especially in settings where health facility stock-out are common, will need more evaluation.

Whether volunteers should be offered incentives to encourage sustainability is debated. Some mass drug delivery systems have been successful without incentives whilst others have employed financial incentives of some kind. In The Gambia, the tradition has been that VHWs should be supported by their community, an approach that has not been particularly successful over the years in many villages, leading to some VHWs becoming disillusioned due to lack of financial and nonfinancial support. In Gambia VHW can be paid US$7.50 per day for participation in mass drug administration, polio, or vitamin A campaigns. In this study, VHWs received an incentive of $11 per month. It is highly likely this financial payment and the strengthened drug supply contributed to the trials success. However, even with a small financial payment the cost effectiveness of IPTc is still comparable to that of other malaria interventions. Any incentives must reach the VHW in a timely and efficient manner to avoid demotivation. Longer term experience will show whether the high level of coverage obtained in this study can be sustained but the results of this trial suggest that community volunteers can achieve high level coverage with this very effective malaria control intervention if they are supported to do so.

## Supporting Information

Text S1 Study protocol.
(0.18 MB PDF)

Text S2 CONSORT checklist.
(0.03 MB PDF)

## Abbreviations

AQ: amodiaquine
AS: artesunate
CI: confidence interval
EPI: expanded programme on immunisation
Hb: haemoglobin
IPT: intermittent preventive treatment
IPTc: intermittent preventive treatment in children
IPTi: intermittent preventive treatment in infants
IPTp: intermittent preventive treatment in pregnant women
ITN: insecticide-treated bednet
RCH: reproductive and child health
SP: sulphadoxine pyrimethamine
URR: Upper River Region
VHW: village health worker
