# Supplementary material for: Two Strategies for the Delivery of IPTc in an Area of Seasonal Malaria Transmission in The Gambia: A Randomised Controlled Trial
Source: PLoS Med. 2011 Feb 1;8(2):e1000409. doi: 10.1371/journal.pmed.1000409 (PMC3032548; doi:10.1371/journal.pmed.1000409)
Supplement: Text S1 — Study protocol. (0.18 MB PDF) [file pmed.1000409.s001.pdf]

Medical Research Council Laboratories, Fajara
Application to undertake a research project

A Summary Information

A1 Title of project

Please choose a short clear title for ease of reference and identification in future.

Comparison of two strategies for the delivery of intermittent preventive treatment in children (IPTc) in an area of seasonal malaria transmission

A2 SCC Number ……………………………(To be allocated)

Is this a resubmission?………………………………..YES ~~NO~~

For ALL submissions and resubmissions a version number and version date needs to be entered below. Resubmissions may not be considered without this information

VERSION NUMBER 03

Date of this version: 18th January 2006

A3 Investigators (Principal Investigator first)

Please list all investigators and collaborators and attach CV if the principal investigator is unknown to the Committee

Name	Institution	Position
Kalifa Bojang	MRC Laboratories	Research clinician
Paul Milligan	London School of Hygiene and Tropical Medicine	Statistician
Momodou Jassey	MRC Laboratories	Demographer
Ayo Palmer	Centre for Innovation against Malaria	Director
Warren Stevens	MRC Laboratories	Health Economist
Harparkash Kaur	Gates Malaria Partnership	Phamacologist
Saikouna Sanyang	URD District Health Team	Head
Malang Fofana	National Malaria Control Programme	Head
Lesong Conteh	London School of Hygiene and Tropical Medicine	Health Economist
Ismaela Abubakr	Malaria Programme, MRC Laboratories	Data manager
Virginia Wiseman	London School of Hygiene and Tropical Medicine	Health Economist
Kebba Gibba	EPI Unit, Department of State for Health	Head
David Conway	Malaria Programme, MRC Laboratories	Head
Brian Greenwood	Gates Malaria Partnership	Director

Who will introduce the proposal at SCC?

Kalifa Bojang

The PI, if present in The Gambia will normally be invited to present the proposal to the meeting

A4 Location(s) of research

Please list all the places where the research will take place including field sites or health facilities

Basse Field Station

A5 Proposed start date and duration in months

February 2006, for two years

A6 Summary of project, long-term objectives and specific aims (not more than 200 words)

This section is very helpful to the Committees in determining quickly the main features of the study, and should be as clear and concise as possible. It should cover the key objectives and endpoints and, if the project is hypothesis driven, then the hypothesis should be stated here.

Antimalarial chemoprophylaxis can reduce morbidity and mortality from malaria in children. However, this approach to malaria control has not been implemented widely because of concerns over its possible effect on the development of resistance and natural immunity. Intermittent preventive treatment (IPT) may be able to achieve some of the beneficial effects of chemoprophylaxis without its drawbacks. Recently, it has been shown that IPT given to Senegalese children under the age of five years on three occasions during the malaria transmission season reduced the incidence of clinical malaria by approximately 90%. However, it is uncertain how this intervention can be most effectively delivered. Therefore, 26 Maternal and Child Health (MCH) trekking clinics in Upper River Division, south of the River Gambia, each with an average catchment population of 400-500 children under 5 years of age, will be randomly allocated to receive IPT from the MCH trekking team or from a IPT dispenser (village health worker, traditional birth attendant or a community mother based in a primary health care village). Treatment with a single dose of sulfadoxine /pyrimethamine (SP) plus three doses of amodiaquine will be given to all study subjects at monthly intervals on three occasions during the months of September, October and November. The primary end points will be the incidence of clinical attacks of malaria detected by passive case detection, and cost-effectiveness of the delivery methods. Important secondary endpoints will be the coverage and the equity of coverage of IPT in preventing malaria morbidity.

A7 Confidentiality

SCC applications will normally be available on the MRC Gambia intranet with access for all senior staff. If for reasons of commercial, ethical or scientific sensitivity you wish to restrict access/circulation to SCC Committee members only please indicate here.

Restrict access to SCC members? ~~YES~~ NO

A8 Checklist/Signatures

Please complete the following checklist and comment as appropriate. This section is designed to ensure that all the planning steps have been taken that are needed for a successful project and that the resource requirements are appropriately laid out in Section D. For projects at the MRC Laboratories, Programme Heads will help visiting workers, and others preparing proposals at a distance, to ensure liaison with key individuals who need to be consulted locally.

a) **Has the project been discussed and cleared with the institutions in which research will be carried out including health services to which the study will need access?** Yes

b) **Have all investigators and collaborators given their agreement to take part in the study as described?** Yes

c) Have ethical issues been addressed? Yes Give details in section C**.**

Does the project require laboratory work, new laboratory procedures, or the riding of motorcycles? Have the safety issues been addressed? (The Health and Safety Manager will advise on risk assessment)

There are no new procedures. MRC laboratory and field safety rules will be followed.

d) Will the project require data and/or materials to be taken out of The Gambia? If so please give details and sign the following statements:

The principal investigator undertakes to leave with the Computer Centre data archivist in The Gambia, a complete copy of the data set at the following two time points:

1. After data entry and verification ("raw data sets")
2. At the point of submission for publication of final report ("analysis data sets")

Signed…………………………………………………..

The principal investigator undertakes to leave with MRC Laboratories The Gambia appropriate aliquots of the biological material being taken out of the country

Signed……………………………………………

e) For projects to be carried out at MRC Laboratories:
Has the project been discussed with the following support staff as appropriate and resource requests agreed? (Give details where relevant in section D) :

	DATE DISCUSSED	Comment
Health and Safety Manager		Will be consulted
Head of IT/Senior Data Manager		Will be consulted
Director of Clinical Services		Will be consulted
Scientific Administrator		Will be consulted
Transport Manager		Will be consulted
Finance Manager		Will be consulted
Personnel Manager		Will be consulted
Director of Operations		Will be consulted
Other services – specify		

Signature of principal investigator: **Date:**

B Description of Project

not more than 5 pages covering the following:

B1 Background

The background should show the relationship between the proposed study and the present state of knowledge and should reference previous work by the investigators and others. The results of any pilot experiments should be stated.

1.1 Introduction

The results of trials in The Gambia and other countries have shown that antimalarial chemoprophylaxis reduced malaria morbidity, school absenteeism, and all-cause mortality[1-5]. However, this approach to malaria control has not been implemented widely due to concerns over its possible effect on the development of resistance, logistic constraints and its potential effect on the development of natural immunity[6-7]. To take advantage of the protective effect of chemoprophylaxis whilst reducing its possible adverse effect on the development of natural immunity to malaria, the concept of intermittent preventive treatment (IPT) with an effective antimalarial has been proposed. IPT involves administration of a full treatment dose of an anti-malarial drug at specific times, regardless of the presence or absence of malaria parasites[8]. Since treatment is only given intermittently, it has been argued that this intervention is less likely to interfere with the development of natural immunity than sustained chemoprophylaxis.

Use of IPT as a malaria and anaemia control strategy was initially explored in pregnant women. Encouraging results from these trials generated interest in the use of IPT for the prevention of malaria and malaria-associated severe anaemia in infants (IPTi). In a study in Tanzanian infants, IPT with sulfadoxine/pyrimethamine (SP) given at the time of routine immunization reduced the rate of clinical malaria by 59% and that of severe anaemia by 50% in an area of moderate transmission where many children slept under ITNs[9]. In a second trial conducted in another part of Tanzania with higher transmission, IPT with amodiaquine provided 65% protection against malaria fevers and 67% protection against anaemia[10]. In Tanzania, protection was sustained into the second year of life, long after drug administration had ceased suggesting that IPT had modulated the immune response to malaria in some way[11]. However, in a further study undertaken in an area of high, seasonal transmission in Ghana a lower level of initial protection was seen and this was not sustained [12]. Further studies of IPTi using SP and other drugs are underway under the auspices of the IPTi consortium[13]. If these studies produce encouraging results, IPTi may be adopted by some countries as national policy.

Unless the long-standing effects of IPTi seen in the Tanzanian study are replicated elsewhere, IPTi is likely to have a major impact on the burden of malaria only in countries where the main burden of malaria is in infants. This is not the case in many countries of the Sahelian and sub-Sahelian regions of Africa with seasonal malaria transmission where the main burden of malaria is in older children. In these countries, there is a need to find additional methods for preventing malaria in older children - IPT provides a possible way of doing this. Two studies carried out in Kenyan children aged 2-36 months with mild to moderate anaemia showed that IPT with SP resulted in modest reduction in incidence of clinical malaria and the proportion of children with anaemia[14, 15]. More dramatic results were obtained in Senegal, where SP and one dose of artesunate given to children less than 5 years old three times, at one monthly interval, throughout the

peak period of malaria transmission season resulted in approximately 90% reduction in clinical malaria[16]. In neighbouring Mali, IPT with two doses of SP led to a 40% reduction in the incidence of clinical attacks of malaria [17]. Artesunate was included in the initial regimen used for IPT in Senegal in the expectation that use of this drug would reduce the emergence of parasites resistant to SP and the prevalence of gametocytaemia, following on a recommendation of the WHO. However, nearly all parasites found in treated children at the end of the transmission season carried markers for resistance to pyrimethamine[16]. Thus, in 2004 a further trial was undertaken in the same study site in 2102 children to compare different treatment regimens. The combinations investigated were SP plus one dose of artesunate, SP plus three doses of artesunate, SP plus three doses of amodiaquine and three doses of artesunate and amodiaquine. The children were visited after each treatment to record any adverse events and were kept under weekly malaria surveillance throughout the 2004 malaria season. The best results where obtained with SP plus amodiaquine. This regimen showed the highest level of protection against infection and the lowest prevalence of parasitaemia at the end of the malaria transmission season. The prevalence of resistance markers to pyrimethamine at the end of the transmission season was high and similar in each group. However, the prevalence of asexual and sexual parasitaemia was extremely low so there was little scope for onward transmission of resistant parasites. The main side effects recorded were fever, vomiting, agitation and headache. Each of these symptoms was seen more frequently in children who took amodiaquine than in children in the SP plus AS groups, but the overall incidence was low[18].

IPTi has a major advantage over IPT in older children in that a delivery system for drug administration, the EPI programme, already exists. A major challenge for IPTc will be finding a delivery system that is cost effective and sustainable. In the studies undertaken in Senegal and Mali, drugs were given under the direction of project staff. In Ghana, a study is exploring the use of community volunteers to give IPTc and this approach appears to be working well in this community (Kweku – personal communication). However, there is a need to explore other ways in which IPTc could be administered effectively and cost effectively in other communities.

1.2 Cost-effectiveness and health inequalities

The study outlined in this proposal will be an evaluation of two different methods of delivery of an intervention which has been shown to be effective in two small studies, conducted in Senegal and Mali, when implemented under the conditions of a closely monitored efficacy trial. The results of the study proposed in this application should help to guide policymakers on how to implement this intervention within a national health care delivery programme and cost-effectiveness will, therefore, be an important end-point for the trial. The study will also address the issue of inequalities in the delivery of a potentially valuable malaria control tool. Health inequalities and inequality of access and utilization of health care are still a huge problem in developing countries[19] and few interventions are directed towards alleviating this. Most causes of inequalities are thought to stem from lack of access through remoteness of location and low socioeconomic status leading to an inability to access funds to travel to health centres and clinics [20]. These subgroups within the population are likely to benefit from an intervention such as IPT which is delivered in the community and does not rely on high levels of health seeking behavior or health service utilization. This study will attempt to measure not only the

relative cost-effectiveness of the two approaches to delivery of IPTc but will also investigate the impact of the two methods on health inequalities in regard to malaria morbidity through a case control study.

1.3 Health care delivery in The Gambia

The components of the Gambian health care delivery system include central referral hospitals, facility-based basic health services (dispensaries, minor and major health centres) and village health services (VHS). Basic health service facilities (BHS) provide technical support for the VHS. The Divisional Health Teams (DHTs) are responsible for supervising and providing supplies for BHS and VHS.

Primary health care villages have usually been selected from among those with a population over 400 and, occasionally, from ones located in relatively isolated areas. In these villages, village health workers (VHW's) and traditional birth attendants (TBA's) are selected by the Village Development Committee (VDC) and are given 6 to 8 weeks of training with a standardised curriculum. VHW's function as primary health care providers for minor illnesses and injuries for all ages. In addition, the VHW is expected to work as a community-based health educator. TBA's function as birth attendants, family planning distributors and health educators. Both TBA's and VHW's are expected to refer serious cases to the local health facility. VHW's and TBA's are supervised by Community Health Nurses who oversee circuits of 4 to 10 PHC villages. CHN's report in turn to their nearest BHS facility and are supervised by the Officer In Charge (OIC) of that facility and by the Divisional Health Team.

1.4 The Expanded Programme on Immunization in The Gambia

In The Gambia, EPI was started in 1979. Initially, yellow fever vaccine and the six "traditional" EPI vaccines (BCG, DPT, oral polio vaccine, tetanus toxoid and measles vaccine) were included in the schedule. Hepatitis B and *Haemophilus influenzae* type B vaccines were introduced into the EPI schedule in 1990 and 1997 respectively, following successful vaccine efficacy trials conducted in the country. The key strengths of The Gambian EPI programme are its high access rate (>90% geographical coverage in 2004), high immunization coverage rate (92% of the children received three doses of DPT in 2004) and high public awareness about EPI vaccines. EPI is implemented through base clinics, which take place in dispensaries, minor and major health centres. The number of base immunization sessions varies from 1 to 5 in a week. Trekking or mobile clinics serve the population, which cannot travel to a base facility. In each basic health facility, the maternal and child health clinic (MCH) teams use vehicles or motorcycles to deliver activities such as EPI vaccinations, administration of vitamin A capsules and registration of births at outreach stations.

1.5 IPTc treatment regimen

Based on the results of the 2004 study in Senegal described in section 1.1 above, we propose to use one dose of SP plus three doses of amodiaquine as the regimen for IPTc in this study. SP is still effective in The Gambia. In 2003, a trial carried out in three sites in The Gambia, showed a day 14 clinical failure rate of less than 2% with SP in combination with choloroquine or amodiaquine when used to treat uncomplicated malaria

cases (Dunyo S B et al, unpublished data). Efficacy trials of artemisinin combinations conducted in The Gambia have shown the combinations to be safe and highly efficacious[21]. The use of SP and amodiaquine rather than an artesunate combination has advantages of cost and allows artemisinin combinations to be reserved for treating acute clinical malaria, among whom their rapid action may be especially helpful.

2. OBJECTIVES

The objective of this trial is to study the effectiveness and cost-effectiveness of two approaches to the administration of intermittent preventive treatment to Gambian children – distribution by village volunteers or through EPI trekking teams.

B2 References

1 Bradley-Moore AK, Greenwood B, Bradley DJ. Malaria chemoprophylaxis with chloroquine in young Nigerian children. *Ann Trop Med Parasitol* 1985; **79:** 563–73.

2 Greenwood BM, Greenwood AM, Bradley AK, et al. Comparison of two strategies for control of malaria within a primary health care programme in The Gambia. *Lancet* 1988; **1:** 1121–27.

3 Alonso PL, Lindsay SW, Armstrong JR, et al. The effect of insecticide-treated bed nets on mortality of Gambian children. *Lancet* 1991; **337:** 1499–502.

4 Menon A, Snow RW, Byass P, Greenwood BM. Sustained protection against mortality and morbidity from malaria in rural Gambian children by chemoprophylaxis given by village health workers. *Trans R Soc Trop Med Hyg* 1990; **84:** 768–72.

5. Menendez C, Kahigwa E, Hirt R, et al. Randomised placebo-controlled trial of iron supplementation and malaria chemoprophylaxis for prevention of severe anaemia and malaria in Tanzanian infants. *Lancet* 1997;**350:**844-50.

6 Laing AB. The impact of malaria chemoprophylaxis in Africa with special reference to Madagascar, Cameroon, and Senegal. *Bull World Health Organ* 1984; **62** (suppl)**:** 41–48.

7 Pringle G, Avery-Jones S. Observations on the early course of untreated Falciparum malaria in semi-immune African children following a short period of protection. *Bull World Health Organ* 1966; **34:** 269–72.

8.Greenwood B. The use of antimalarial drugs to prevent malaria in population of malaria-endemic areas. *Am J Trop Med Hyg* 2004; 70: 1-7

9.David Schellenberg, Clara Menendez, Elizeus Kahigwa, et al., Intermittent treatment for malaria and anaemia control at time of routine vaccinations in Tanzanian infants: a randomised, placebo controlled trial. *Lancet* 1997; **357**: 1471-77

10.Massaga JJ, Kitua AY, Lemnge MM, et al., Effect of intermittent treatment with amodiaquine on anaemia and malarial fevers in infants in Tanzania: a randomised placebo-controlled trial. *Lancet.* 2003 ;**361**:1853-60.

11. Schellenberg D, Menednez C, Aponye JJ, et al. Intermittent precventive antimalrial treatment for Tanzanian infants;follow-uo to age 2 years of a randomised, placebo-controlled trial. *Lancet* 2005;**365**:1481-83.

12. Chandramohan D, Owusu-Agyei S, Carneiro I et al. Clkusrter randsomosed trial of intemrittnet preventive treatment for malaria in infants in area of high, seasonal; transmission in Ghana. *Brit Med J* 2005;**331**:727-33.

13. Intermittent Preventive treatment in Infants Consortium. http://www.ipti-malaria.org.

14. Desai MR, Mei JV, Kariuki SK, et al., Randomized, controlled trial of daily iron supplementation and intermittent sulfadoxine-pyrimethamine for the treatment of mild childhood anemia in western Kenya. *J Infect Dis*. 2003 ;187:658-66

15. Verhoef H, West CE, Nzyuko SM, de Vogel S, et al., Intermittent administration of iron and sulfadoxine-pyrimethamine to control anaemia in Kenyan children: a randomised controlled trial. *Lancet.* 2002 ;360: 908-14.

16. Cisse B, Sokhna C, Boulanger D et al. Seasonal intermittent preventive treatment with srtesunate and sulfadoxine-pyriemthamine prevents malaria in Senegales children. Lancet, in press.

17. Dicko A, Sagara I, Sissoko MS et al. Impact of intermittent preventive treatment with sulfadoxine-pyriemthamine targeting the transmission season on the incidence of clinical malaria in children 6 months to 10 years in Kambila Mali. Abstract. *Am J Trop Med Hyg* 2004; 71 supplement. 6.

18. Sokna et al. in preparation

19. Davidson R Gwatkin, Abbas Bhuiya, Cesar G Victora. Making health systems more equitable. *Lancet* 2004; **364**: 1273-80

20. Filmer D, Pritchett L. The impact of public spending on health: does money matter? *Soc Sci Med.* 1999 ;49 :1309-23.

21. von Seidlein L, Milligan P, Pinder M, et al., Efficacy of artesunate plus pyrimethamine-sulphadoxine for uncomplicated malaria in Gambian children: a double-blind, randomised, controlled trial. *Lancet.* 2000; 355: 352-7.

22. Greenwood BM, Bradley AK, Greenwood AM, et al. Mortality and morbidity from malaria among children in a rural area of The Gambia, West Africa. *Trans R Soc Trop Med Hyg* 1987;**81:** 478-486.

23. Green A. (1999). An Introduction to Health Planning in Developing Countries. Oxford University Press, Oxford.

24. Beyai P.L. (2005). Exploring the measurement of indirect costs via an economic evaluation of Intermittent Preventive Treatment for pregnant Gambian women.
Thesis submitted to the Faculty of Medicine of the University of London for the degree of Doctor of Philosophy.

25. DeMTAP study (2005). Gates Malaria Partnership Annual Report 2005.
http://www.lshtm.ac.uk/gmp/annualreport.htm

B3 Project description

This should cover project plan, time-scales, descriptions of methods, justification, analyses to be carried out, expected outcomes (see also B4 and B5 where particular details need to be set out and cross refer as necessary).

B STUDY DESIGN AND METHODOLOGY

3.1 Study area

The study will be conducted in the part of the Upper River Division (URD) south of the River Gambia. There are 3 basic health facilities in this area: Gambisara Dispensary, Basse and Fatoto Health Centres. In-patient facilities are available in Basse and Fatoto Health Centres. All the three basic health facilities operate monthly trekking clinics to 27 outreach locations for delivery of EPI vaccination, administration of Vitamin A capsules to children 6-11mths, and registration of births. In the South Bank area of the URD there are 7 Key Primary Health Care (PHC) villages each serving a number of satellite non-PHC villages. Each key PHC village has a health post staffed by a Community Health Nurse. The majority of PHC villages have a Village Health Worker although not all are functional. There are 29 PHC villages in the study area.

3.2 Enumeration, recruitment and consent

The study will be carried in collaboration with the EPI unit of the Department of State for Health, National Malaria Control Programme and URD District Health Team. During the preparatory phase of the study, the design and objectives of the study will be discussed extensively with the URD District Team and all health care providers working in the study area. Between February and March 2006 the study will be publicized on Radio Basse. There is a regular health programme slot every Tuesday at 8.30 PM. In addition, the study team will visit all the villages in the study area to explain the objectives of the trial to village elders, opinion leaders, heads of women's groups, village health workers and traditional birth attendants. This will be followed by village meetings, which the entire community will be invited to participate and villagers will be asked to identify one person who will be responsible for distribution of the trial medication in the village (designated as an IPTc dispenser). This person could be a village health worker, traditional birth attendant, a community mother or any respectable person in the village. During these meetings, agreement to participate in the study will be obtained from the villagers. In addition, parents or guardians of prospective study subjects in the right age group will be visited at home and they will be provided with flyers in English and the appropriate local language (Mandinka, Sarahule or Fula). During these visits, a reporter will be identified in each village and he will be asked to keep records of all births, and all deaths, immigrations and emigrations of children under five years of age during the study period. The village reporter will be paid a modest salary for his contribution to the project per event recorded. Village reporters will be visited fortnightly by project field workers and their records collected and checked. Deaths will be reported to a field supervisor who will be based in the study area. Deaths will be investigated using the post-mortem questionnaire techniques and cause of death established whenever possible.

Enumeration of all children under 6 years of age in the study area will be carried out between March -May 2006 and a database established before the start of the study. Written informed consent will be obtained from the parent(s) or guardian(s) before a child is enrolled in the study (see annex). Approximately 12000-14000 children will be eligible for enrolment in the study. Enrolled subjects will be provided with a “malaria card” (described below) stamped with a label carrying their name and study number to facilitate identification at each contact. GIS done for the south bank of URD during the pneumococcal vaccine trial (PVT) will be updated and this will be used to identify study households.

The unit of randomization will be the catchment population of the monthly EPI trekking clinics. There are 33 trekking clinics in total on the south bank of URD of which 27 are rural. The rural trekking clinics will be selected for randomization. 26 rural clusters (two will be merged to give an even number) will be stratified by health facility catchment area (Gambissara, Basse, Fatoto). Constrained randomization will be used to ensure balance within strata with regard to distribution of PHC key villages and population size.

The bednet coverage in URD is estimated to 65%. However, during the enumeration details of bed net usage by the community will be assessed. In collaboration with the malaria control programme, we will facilitate the provision of bed nets to households without one. At the beginning of the rainy season permethrin will be provided to all households with bednets.

3.3 Malaria card

After the enumeration has been completed, a database containing the list of eligible children will be established. The database will be used to generate the list of participating study children in each village. A record system using appropriate visual aids will be devised that can be used by an IPTc dispenser. This will consist of a “malaria card” held by the mother or guardian of each child and an IPT register held by IPTc dispenser or staff of the DHT trekking team. On the malaria card held by the mother, the compound number and study number will be written in Arabic and English, together with the child’s name and the name of his or her parents. The dosage of trial medication to be given will be indicated on the malaria card and the register using coloured circles and semi-circles (full circle for one tablet and semi-circle for half a tablet). The register held by the IPTc dispenser or DHT staff will contain similar information as that on the malaria card.

When a child presents to a health facility for medication, the DHT staff or the IPTc dispenser will examine the malaria card of the study subject. Medication will only be given to study subjects after correct identification and after matching the information on the malaria card held by the mother and that on the register held by the IPTc dispenser or the DHT. It is envisaged that the DHT staff and IPTc dispenser taking part in the study will be paid modest salary supplements for their contribution to the project. However, the details of payment will be discussed with the EPI unit and Basse DHT.

3.4 Training of village-based IPTc dispenser

The person who will be responsible for the distribution of trial medication in the study villages will receive a period of training on how to distribute monthly preventive treatment to children and how to maintain a compliance ledger.

3.5 Cross-section survey

In December, at the end of the malaria transmission season, a cross-sectional survey of children less than 6yrs will be undertaken in all the 26 clusters. We propose to sample 40 children per cluster in all 26 clusters, a total of 1014. A questionnaire to determine asset rating and wealth index will be completed. Information on demographic, **bed net usage,** socio-economic status, health seeking behaviour and utilisation patterns profiles will be collected, a clinical history will be obtained and a physical examination will be performed, including abdominal palpation for splenomegaly and measurement of height, weight and axillary body temperature. In 12 clusters (6 from each arm, identified at randomization) a finger-prick blood sample will be obtained from 40 children per cluster for preparation of blood films, preparation of a filter paper sample and determination of the haemoglobin level. During the survey, febrile children will be screened for malaria using a dipstick rapid antigen test. Study subjects with documented fever (axillary temperature of ≥ 37.5°C) or a history of recent fever and positive antigen test will be treated with coartem. Study patients needing admission will be referred to Basse Health Centre or Bansang Hospital.

3.6 Distribution of tablets

3.6.1 Distribution of tablets in the villages

There are seven key villages in the study area, each serving a number of satellite PHC villages. Each key PHC village has a health post staffed by a community health nurse. There are 29 PHC villages and the majority of these have a village health worker and a traditional birth attendant. The three towns with basic health facilities (Basse, Fatoto and Gambisara) will be excluded from the evaluation, and the trial will focus on other communities in the study area. The trial medication will be administered by IPTc dispenser based in a PHC village. The IPTc dispenser will distribute the trial medication at a central point (usually a health post) on the first Wednesday of the month during September, October and November. Wednesday is chosen, as women usually stay at home on that day rather than work in the fields. Mothers and carers will be asked to bring their children to the central point during the morning when the IPTc dispenser will be available to distribute the drugs. When a child presents to the central point for medication, the IPTc dispenser will identify the study subject using the malaria card held by the mother and match the information on the malaria card with that on her register. When she is satisfied that she has correctly identify the study subject and that the information on the malaria card matches those on her register, the correct dosage of the trial medication will be given to the study subject. In non-PHC villages, mothers will be asked to bring their children under 5 years of age to their nearest PHC village to receive IPT during the transmission season.

The first dose of treatment will be taken under direct supervision of the IPTc dispenser and she will mark her register and the malaria card to show that the child has received the tablets. The remaining two doses will be given to the mother or guardian of the child with

clear instructions as how to administer the drugs. The IPTc dispenser will not administer treatment to sick children enrolled in the study; instead she will refer them to the nearest health centre for management, and indicate referral on the malaria card and register. One field assistant will be based at each key PHC village and he will visit each PHC village regularly. His main role will be to facilitate the referral of study subjects to the nearest health centre and provide supplies to IPTc dispensers

To ensure that drugs supplied to the IPTc dispenser are dispensed to study subjects in accordance with the protocol, only a-month's supply of drugs will be provided for each round of treatment and the drugs will be stored in secured boxes. In addition, an audit will carried out at regular intervals. During these audits, study drug delivery records will be reconciled with those of usage and returned stocks and any discrepancies found will have to be accounted for by the IPTc dispenser. During training of the IPTc dispenser, the need to use the drugs only for the intended purpose will be emphasized.

3.6.2 Distribution of tablets in the trekking clinics

A member of the MCH trekking team (designated as an IPTc officer) of each of the 3 basic health facilities (Basse, Fatoto and Gambissara) will be identified by the EPI team and given responsibility for delivery of IPT at the trekking clinics, which are allocated, by randomization, to deliver IPTc. Thus, each EPI team will deliver IPTc in some of its trekking clinics but not in others. A project nurse and a field worker will be attached to each trekking team to identify children in the trial and to ensure that they receive their monthly trial medication. The trekking team will be provided with a list containing the study number , village and name for each study subject scheduled to attend that trekking clinic. The first dose of treatment will be taken under direct supervision of the IPTc officer and he will mark his register and the malaria card to show that the child has received the tablets. The remaining two doses will be given to the mother or guardian of the child with clear instructions as how to administer the drugs.

The dates of each monthly treatment will be written in Arabic and English on the "malaria cards". To ensure that children do not receive more doses of IPTc than they should, different coloured "malaria cards" will be issued to children in villages where children will receive their medication from IPTc dispenser and to those allocated to receive medication from the MCH trekking clinic. IPTc dispensers and IPTc officers will be taught how to give medication to children who have only the correct coloured cards.

At the end of the malaria transmission season, the total number of complete treatment doses each child in the trial has taken will be recorded. Compliance will be checked on a rolling basis throughout the study by assessing the number of correct doses of medication received. In addition, urine samples will be collected from a random sample of 100 children each month in the two-week period following administration of IPTc to test for the presence of SP in the urine using Eggelte dipsticks.

3.7 Accuracy of recording of treatments in the two arms of the trial

Throughout the study, the village and EPI team records of treatments will be checked against the malaria cards to ensure accuracy and completeness and to check that there is no difference in the quality of record keeping in the two study arms.

3.8 Contamination

Spot checks will be made throughout the study to assess whether children in villages randomized to EPI delivery receive any treatments from nearby villages with village-based delivery, and vice versa. The importance of avoiding treating children from outside the cluster will be emphasised to the community workers and EPI teams. Malaria cards will bear a clear cluster identifier to facilitate this.

3.9 Morbidity surveillance during the rainy season

Passive surveillance for malaria will be maintained throughout the transmission season. Parents/guardians will be encouraged to take their child to the health centre identified as being closest to their home at any time their child becomes unwell. Project staff will be based at each of these health facilities to identify children in the trial and to ensure that they are seen, properly investigated and treated promptly. At each clinic visit axillary temperature will be recorded using a digital thermometer and the haemoglobin concentration measured using a Hemocue machine. A dipstick for diagnosis of malaria will be used if fever (axillary temperature of ≥ 37.5°C) or a history of fever within the previous 48 hours is present. In such cases, a thick blood smear will also be collected for subsequent confirmation of the diagnosis. Study subjects with documented fever (axillary temperature of ≥ 37.5°C) or history of recent fever and malaria parasitaemia will be treated with coartem. The treatment of study subjects seen at the health centres for other conditions will be the carried out in accordance with national guidelines.

Denominators for determination of malaria incidence rates will be determined from the census, updated according to information on deaths and emigrations from the village recorders.

3.10 Surveillance for severe malaria

Records will be kept of admission of study children to Basse and Fatoto Health Centres. Physicians and nurses based at these facilities will be asked to look out for any child with severe malaria, and any admission attributed to malaria will be carefully documented.

3.11 Surveillance for overall and cause specific mortality

Deaths will be investigated using post-mortem questionnaire techniques and cause of death established wherever possible. The health card will be used to confirm the identity of the deceased child and may provide some important information about the health and nutritional status of the child prior to death.

3.12 Nested case control study

To estimate the effect of inequalities in wealth on malaria morbidity and its prevention by intermittent treatment, -a nested case control approach will be used to determine distribution of malaria morbidity in relation to two measures of socioeconomc status - a wealth rating scale and an asset index. The case control study will also allow efficacy of IPT to be estimated. Each time a case of malaria is detected, the home of the case will be visited to complete the wealth and asset questionnaire, to record the number and dates of IPT doses received, **whether the child slept under a bednet at the time she or he became sick and the condition (intact, impregnated in the last 12 months) of the bednet.** Controls without evidence of febrile illness will be sought from the same cluster, selected by simple random sampling from the master list of children in the census and the same information collected from them as from the cases. (Matching by study arm is required since we wish to look at interactions with delivery method; matching by cluster is proposed since malaria incidence/access to health centre, IPT coverage, and socioeconomic status are all likely to vary appreciably from cluster to cluster). Four controls will be chosen per case . Interviewing of controls will take place soon as possible after the presentation of a case. As this is an incident-cases study, the odds ratio will provide an estimate of the incidence rate ratio of malaria according to the exposure variables (wealth and asset scores and IPT doses received). As well as looking at main effects of the exposure variables, there is interest in the interaction of intervention delivery type and wealth or asset index, in order to see if one intervention achieves a more equitable distribution, and in the interaction of IPT efficacy with time since last IPT dose, to see how quickly the protection wanes and with bednet use.

3.13 Study of cost-effectiveness

A cost-effectiveness analysis will be undertaken from a societal perspective, meaning that all costs and effects, no matter to whom they accrue, will be measured and valued.

3.13.1 Programme costs: The costs of the two modes of delivering IPTc will be assessed using the bottom-up or ‘ingredients’ approach which involves the identification of all the resources required by the two modes of delivery[23]. Costs will be categorised into those borne by the health service and those by patients and their families. Both the recurrent and capital costs to the health service of training staff and delivering the intervention will be estimated. Both direct and indirect costs to users of the services (i.e. children and their families) will also be assessed. These include any charges or fees, transport costs or time away from work or home. These data will be collected through reviews of hospital records and accounts as well as interviews with key spokespersons from the health services.

3.13.2 Resource savings : The modes of delivery will be compared with respect to the resource savings resulting from fewer children being treated for malaria related illnesses as a result of better IPTc coverage. Data on inpatient and outpatient treatment costs will be collected using questionnaires recently developed for another IPT study in The Gambia[24]. For the health service, interviews will be conducted with facility officers in the

study area and in referral facilities. Administration records at these facilities will also be reviewed. Potential savings to users such as reduced expenditure on transport and other out of pocket expenses and potential savings in time lost from seeking treatment will be collected from a random sub sample of 15 participants attending each of the 26 maternal and child health clinics (n= 390). The difference between delivery modes in total savings will be taken into account when comparing the net cost of the two delivery methods

3.13.3 Adverse effects on EPI delivery: To investigate whether IPT impairs delivery of EPI, indicators of EPI delivery will be collected (e.g. number of doses delivered) in both arms of the trial.

3.13.4 Cost-effectiveness: For both modes of delivery, two cost-effectiveness ratios will be calculated: (i) cost per child who fully adheres to treatment; (ii) cost per case of malaria averted. In addition, *net* cost-effectiveness ratios will be calculated by subtracting resources saved from the total programme cost divided by the relevant outcome measure. The incidence of malaria and the costs will be compared between the two delivery arms and the cost per additional case averted calculated.

3.14 Equity

The impact of the different delivery strategies on alleviating (or exacerbating) three forms of equity will be measured. Equity outcomes and the cost of each delivery mode will also be compared using benefit-incidence analysis.

3.14.1 Equality of health: The impact of the two strategies for delivering IPT on health inequalities will be measured in terms of reduction in cases of malaria morbidity. The aim is to identify the extent to which groups in society are potentially able to benefit from the two interventions and, in turn, whether one mode of delivery is more effective at alleviating malaria burden in highly vulnerable or needy groups. A case control approach (see section 3.12) will be used. Two methods will be used to assess socio-economic status of cases and controls; (i) asset index recently used in The Gambia[25] and (ii) a wealth rating scale. For both delivery strategies, the socio-economic profile of participants who declined to take part in the study will also be investigated.

3.14.2 Equality of utilisation or adherence: to determine impact in terms of IPT coverage, levels of adherence will also be compared across the two delivery strategies; a cross-section survey will be used. For both delivery strategies, inequalities in utilisation and adherence will then be measured across socio-economic groups and by household location in a cross-sectional survey at the end of the intervention (see section 3.5).

3.14.3 Equality of access: here we are measuring whether households have the same opportunity to benefit from each mode of delivery. This involves defining and measuring financial access, geographical access, informational access and finally, cultural/gender barriers to access. Approximately 25 compliers and 25 non-compliers from a sub sample of 12 clusters will be interviewed about factors influencing access to each delivery strategy. Within each cluster, participants will be purposively sampled to ensure that people of different ethnicity, age, gender, education and socio-economic status are

equally represented. Wherever possible both parents will be interviewed. The reason for this is that we expect access barriers to vary between males and females.

3.14.4 Benefit-incidence-analysis: utilisation and access data for rich and poor households will be compared with the cost of providing the service. This will involve the use of benefit-incidence-analysis to analyse the impact of government expenditure on poverty.

3.14.5 Cost-effectiveness data collection

Health service costing data including capital costs, consumables, salaries etc. will be collected by careful review of health facility records and through consultation with administration staff. Costs to the community will be collected using an interviewer administered questionnaire recently developed by a GMP PhD student investigating the cost-effectiveness of IPT in pregnancy in 2004-2005 in The Gambia. The questionnaire will be modified and piloted on different groups of respondents (e.g. different age, gender and ethnic groups) to ensure its relevance to this study population.

3.15 Assessment of resistance to antifolates

Polymerase chain reaction on extracted DNA will be used to test for mutations in *dhfr/dhps* genes from filter paper blood samples collected from all malaria cases detected during the cross-sectional survey and morbidity surveillance.

3.16 Assessment of adverse events

3.16.1 *Eliciting and documenting adverse experiences*

All adverse events or reactions which might be related to drug administration which occur during the trial will be documented. The nature of each experience, date and time (where appropriate) of onset, duration, severity and relationship to treatment will be established whenever possible. A sample of 200 children will be visited on day 3 after treatment to record compliance with the daily doses and any adverse reactions, in addition to a survey of mothers' perceptions of IPTc undertaken during the December survey. A sample of 200 children would allow us to estimate an incidence of adverse events of 5% to within +/-3%; if 200 children are visited in each arm compliance, which may depend on the explanations given to the mother at time of treatment, can be compared between delivery arms.

3.16.2 Serious Adverse Events

A serious adverse experience is any event which is fatal, life threatening, disabling or incapacitating or results in hospitalisation, prolongs a hospital stay or is associated with congenital abnormality, cancer or overdose (either accidental or intentional). Any serious adverse event that occurs within 24 hours of administration of intermittent treatment will be reported to the Local Safety Monitor, Chairman DSMB and director of GMP

3.17 Study end-points:

3.17.1 Primary end-points

The trial will have two primary end-points.

- Malaria incidence (the number of OPD attendances with clinical malaria that meet the case definitions as indicated below during the surveillance period) and the number of hospital admissions with malaria during the surveillance period.
- Cost-effectiveness of the delivery system.

3.17.2 Secondary end-points

- Coverage with IPTc as measured by the following indicators:
  - the proportion of children who received three IPT courses on schedule;
  - the proportion of children who received partial or off-schedule IPT courses
  - the proportion of children with no IPT.
- Unit cost of delivery per fully adherent child.
- Incremental cost-effectiveness ratio for each systems of delivery.
- Mean Hb (g/dl) at the end of malaria transmission.
- Prevalence of malaria parasitaemia at the end of the malaria transmission season.

3.18 Age interaction within clusters
To take account of the effect of age on study endpoints, study subjects will be stratified into 3-12 months, 12-30 months and > 30 months age bands during analysis.

B4 Details of study design and investigations

This section is designed to give the Committees sufficient information to see clearly and quickly the scientific and ethical aspects of the study design. Some parts will not be relevant to all studies. For studies at MRC Laboratories, the please discuss data management arrangements with the Head of Computing. For clinical studies, please discuss the clinical service commitments with the Clinical Director. If you have questionnaires or consent forms prepared, please attach these to the application.

a) What type of study design is proposed (eg case control, prospective cohort, randomised controlled trial, etc)

Cluster randomized and nested case control

b) What is the proposed size of the study (this may relate to patients, cases, controls, survey subjects, laboratory samples etc, as appropriate).

26 trekking clinic with an average catchment population of 400-500 children under 5 years will be randomly allocated to receive IPT from MCH trekking clinic or from a village based IPTc dispenser

c) Please describe the statistical considerations and sample size calculations involved in determining the size of the study. (If you do not have access to statistical advice, please consult the MRC Laboratories Statistics Department.)

The total number of children in the study communities eligible to receive IPT from either community workers or though the EPI clinic will be in the region of 12 – 14,000.

Incidence of malaria

The average number of children 3 months to 5 years old in the catchment area of each trekking clinic is 400-500. Assuming that 75% coverage of three doses of IPT is achieved in both arms, and assuming that the efficacy of IPT is 90% (incidence of malaria reduced by 90%), and assuming that the incidence rate of malaria by passive case detection among children under 5yrs of age is 0.2 per child per transmission season without IPT, then the expected percentage of children with a malaria attack is 0.2*(1-0.9)*00.2*(1-0.9)*0.75 + 0.2*0.25 = 6.5%. To have 90% power that the upper 90% confidence limit for the difference between malaria incidence in the two arms of the study will be less than 0.035, a sample size of 700 children per arm is needed in an individually randomized trial. Assuming malaria incidence in each cluster ranges from 0 to 10% or 0 to 20%, the intraclass correlation (ICC) is roughly 0.01 to 0.04. Although the ICC is small, the large cluster size means the design effect is large. With 26 clusters, the cluster size of 500 per cluster corresponds to 90% power for a non-inferiority margin of 0.035 (if the ICC is 0.01) or 0.05 (if the ICC is 0.04).

Haemoglobin at the end of the malaria transmission season

To calculate sample size for the comparison of haemoglobin concentration between groups at the end of the malaria transmission season, the standard deviation in haemoglobin concentration among children under 5 years in December, the magnitude of the difference in mean Hb between groups that is consider to be important and the cluster-to-cluster standard deviation in the mean Hb need to be taken into account.. The SD of the day 28 Hb among children 6mths to 5 yrs included in a recent Gambian trial of Lapdap was 1.7 g/dl. The SD in children <5 years old seen in December in the Niakhar IPTc study undertaken in 2004 was 1.6 g/dl. However, in this study children who had an Hb<9 g/dl (about 25% of children) when seen in September were given iron so the December SD could be an under-estimate. If the mean Hb ranges between clusters from

9.5 to 11.0g/dl, the SD is about one quarter of the range i.e. 0.375.With an SD within cluster of 1.7 this gives an intraclass correlation (ICC) = 0.375^2/(.375^2+1.7^2) = 0.05, with the SD within of cluster of 1.3 the ICC = 0.08. In the 2004 Niakhar study in which a mean of 150 children were studied in 14 villages in December, the pooled estimate of the within-village SD was 1.64 and the village means ranged from 9.3 to 10.9, with an SD of 0.42. The ICC was 0.04 (95%CI 0.0006 to 0.081). Assuming a normal distribution with SD 1.7 and mean of 10g/dl, a rise of 0.5, 0.75, and 1g/dl corresponds to a 40%, 56% and 67% reduction in the proportion of severely anaemic (Hb<8 g/dl), so the study needs to be powered to detect differences of 0.5 to 0.75 g/dl. between groups. A difference of 0.75g/dl corresponds to 0.44-0.57 SD which is a medium effect size in Cohen's classification. A difference of 0.5g/dl corresponds to 0.29-0.38 SD which is a small to medium effect size in Cohen's classification.

For an equivalence, cluster-randomized study, the sample size can be determined by calculating the number required in an individually randomized trial and then multiplying this by the design effect. If the important difference is 0.75 g/dl, in an individually randomized study a sample size of 89 per group is needed to have 90% power to demonstrate equivalence within a margin of 0.75g/dl, if the SD is 1.7. If the SD is 1.3 this drops to 53 per group and if the power is 80% and the SD 1.7 the number needed is 65. If the important difference is 0.5 g/dl, in an individually randomized study, a sample size of 200 per group is needed to have 90% power to demonstrate equivalence within a margin of 0.5g/dl; if the SD is 1.7. 144 per group are needed for 80 % power. These sample sizes must be inflated to allow for cluster randomization. Using the fact that the design effect Deff=1+(b-1)ICC where b is the number of children sampled per cluster, and b=2nDeff/c, where n is the number per arm needed in an individually-randomized study and c is the total number of clusters, then the number of children to sample per cluster is given by b=2n(1-ICC)/ (c-2.n.ICC). The number of clusters must exceed 2.n.ICC. The table below shows the sample size requirements in the face of different assumptions

Table 1. Sample size for haemoglobin determination.

SD	delta	power	n	ICC	No of subjects per cluster if 26 clusters	Total	No of subjects per cluster if 12 clusters	Total
1.7	0.5	90%	200	0.08	n/a	n/a	n/a	n/a
1.7	0.5	90%	200	0.04	39	1014	n/a	n/a
1.7	0.5	80%	144	0.08	90	2340	n/a	n/a
1.7	0.5	80%	144	0.04	20	520	n/a	n/a
1.7	0.75	90%	89	0.08	14	364	n/a	n/a
1.7	0.75	90%	89	0.04	10	260	36	432
1.7	0.5	80%	65	0.08	8	208	75	900
1.7	0.5	80%	65	0.04	7	182	19	228

If 40 children are sampled per cluster in all 26 clusters, a total of 1014, this will give 90% power for a margin of 0.5g/dl if the ICC is 0.04, and will also allow reasonable power for a difference of 0.75g/dl for subgroup analyses. If Hb sampling is limited to 12 clusters (6 per arm) to simplify fieldwork, a sample of 40 per cluster will allow 90% power for a margin of 0.75g/dl if the ICC is 0.04.

IPT coverage endpoints

Three different indicators of IPTc coverage will be analysed: the proportion of children with no treatment courses, the proportion of children with partial or off-schedule treatment courses, and the proportion of children with three treatments courses received on-schedule. In an individually randomized trial, assuming coverage of IPTc of 50%, 70% or 90% in each arm, then the sample size in each group (n) needed to establish non-inferiority within a margin of 15%, 12% or 10% is as indicated in Table 2

Table 2. Sample size calculations for IPTc coverage levels.

Coverage	Margin	Power	n	c	ICC	Number per cluster
50%	15%	90%	140	26	0.08	72
70%	15%	90%	120	26	0.08	33
90%	15%	90%	56	26	0.08	7
50%	12%	80%	150	26	0.08	139
70%	12%	80%	130	26	0.08	47
90%	12%	80%	60	26	0.08	7
50%	10%	80%	220	26	0.08	n/a
70%	10%	80%	190	26	0.08	n/a
90%	10%	80%	85	26	0.08	13

Thus, if the sample size is 120 per group and expected coverage is 70% (0.7) in both arms, the power is 90% that the upper limit of the observed one-sided 90% confidence interval will be less than 0.15. These sample sizes must be inflated to allow for cluster randomization as was done for Hb, using an estimate of the ICC. If the average coverage is 75% with a range cluster to cluster from 50% to 100%, the standard deviation is approximately a quarter of the range, i.e. 0.125, the coefficient of variation is 0.125/0.75=0.167, and the intraclass correlation is 0.167^2x.75/(1-.75)=0.08. The median of ICC estimates for vaccination coverage of different vaccines in The Gambia found during a coverage survey was 0.088. With c=26 clusters, and ICC=0.08, a sample size of 33 per cluster will give 90% power that the upper limit of the one-sided 90% confidence interval will be less than 0.15 (if coverage is 70%), if 40 are sampled per cluster this will allow for loss due to missing values etc.

Nested case control study.

With 14000 children under 5 years old in the study area, assuming an incidence of malaria by passive case detection of 20%, IPT coverage of 80% and efficacy of 90%, we

anticipate 336 malaria cases. To detect an odds ratio of 2, comparing for example the lower 1/3 of the asset/wealth rating distribution to the upper 2/3, with 80% power (5% significance level), 70-80 matched sets are required. For the test of interaction the sample size is 4 to 5 times greater (Breslow and Day vol 2 p.311 Fig 7.4), so if all available cases are recruited there should be reasonable power to examine this interaction. Similar calculations show that a similar number are required for analysis of IPT efficacy, relatively few matched sets are needed to estimate overall efficacy, but the larger number are needed to look at efficacy in relation to time since dose. Provided the number of cases in each study arm is similar, matching slightly improves the

For studies involving human subjects:

d) How and where will the study subjects (cases, controls, etc) be selected? Has it been confirmed that they are not already involved in other studies?

The study will be based in South Bank of URD. The 27 trekking clinics each with an average population of 400-500 children under 5 years will be selected to take part in the study.

e) What inclusion/exclusion criteria will be applied?

Inclusion criteria

1. Age between 3 months and 5 years at enrolment.
2. Informed consent obtained from parents or legal guardians.
3. No current participation in another malaria intervention trial.

Exclusion criteria

1. Previous adverse reaction to treatment with SP or amodiaquine. If this is unknown, then a history of allergic reaction to any drug.

Withdrawal during the study

Children will be withdrawn from the study only if consent is withdrawn. If there is an adverse event attributed to the study drug, the child will continue to remain in the study but will not receive any further IPT treatment.

f) What samples, if any, will be taken and what investigations will be conducted?

A finger-prick sample will be collected from study subject for malaria diagnosis when they present to health center with symptoms suggestive of malaria. In addition, finger-prick blood samples will be collected from 1014 children selected to take part in the December cross-sectional survey for the malaria thick blood smear preparation and hemoglobin level measurement. Urine sample will be collected from a random sample of 100 children monthly during September, October and November to test for the presence of SP.

g) **Will treatment be given?** **YES** ~~**NO**~~
If yes:
Nature of treatment(s)

For drugs: dosage and duration of treatment

Treatment will be given during the months of September, October and November as follows: SP (tablets containing 500 mg sulfadoxine/ 25 mg pyrimethamine) will be given at an approximate dose of 1.25 mg pyrimethamine/25 mg sulfadoxine per kg and amodiaquine (200 mg base tablets) at an approximate dose of 25 mg/kg over 3 days (10mg/kg D1, 10mg/kg D2 and 5mg/g D3) will be given monthly. Coartem (tablet containing 20 mg artemether and 120 mg lumefantrine) will be given at the following doses: < 15kg: one tablet at the time of diagnosis, and then at 8, 24 and 48 hours.16-25kg : Tow tablets as a single dose at the time of diagnosis, and then at 8, 24 and 48 hours.

Person(s) responsible for administering treatment

Staff of District Health Team or IPTc Dispenser

h) **For questionnaires/interviews, who will be conducting these?**

Field workers

B5 **Data management and Statistical analysis**

a) **Who is responsible for the statistical design and analysis of the study?**

Kalifa Bojang and Paul Milligan

b) **Who will be primarily responsible for database design and data management?**

Ismaela Abubakr

c) **Will data be double entered and verified?**

Yes

d) **The MRC Laboratories IT/Data Management section supports Microsoft Access as its database package. If you are planning to use something else, please indicate which package and give a brief rationale:**

Access will be used for data management

B6 **Expected outputs and Dissemination of results**

(a) **What are the expected outputs(publications) from this project?**

We expect this work will generate at least one scientific paper

(b) What other arrangements will there be to disseminate the findings?

Results will be presented at an international scientific meeting and a report will be submitted to The Gambia Government and Gates Malaria partnership

C. **Ethical issues**

This section is particularly important to the Ethics Committee. Please consult the guidance notes for preparation information sheets and consent forms; the checklist for subject information sheets; the template consent form; the guidelines for scientists (EC); the guidelines for the Gambian initiative for DNA Collections, as appropriate.

(a) Outline how the study will contribute to improving the health of people of The Gambia

Malaria and anaemia are major public health problems in sub-Saharan Africa. IPT offers the possibility for an inexpensive strategy for reducing childhood morbidity and mortality from malaria and anaemia.

(b) Summarise the potential risks and benefits to individuals, communities or country

Only those children whose parents give informed consent will be enrolled. The study involves fingerpick blood samples from febrile children as part of routine patient care. In addition, one further finger prick sample will be obtained from a random sample of 1014 children enrolled in the trial at the end of malaria transmission season.

The study investigates an intervention appropriate to health need of The Gambia.

The drugs used for the trial are safe and have been used in The Gambia

Entry into the trial will not affect the health care the volunteer receives

Trial participants will receive free medical treatment during the study.

(c) How will informed consent be obtained?

The study will be explained in the parents/ guardians' preferred language, and written informed consent will be obtained on approved forms before the start of the study in a non-coercive manner

(d) How will you ensure confidentiality of the data gathered?

Only the study personnel will have access to the data collected and all data collected will be kept in locked cabinet in a secure room with locks. Dedicated computers will be used for data entry and only the PI, data entry clerks and the

data manager will have access to these computers. The computers will be password protected.

(e) Is a consent form attached? Yes

(f) Is a subject information sheet attached? Yes

(g) Is the questionnaire (if applicable) attached? No

D Resources Requested

D1 Summary and cost

The Committees need to be reassured that the resource implications of the study have been fully considered and that the resources are available or are being sought to complete the study. Please refer to the following guidance in completing the table overpage.

For all cost categories please indicate whether (i)internal funds are requested, (ii) whether you plan to vire uncommitted funds in an existing budget (if so please give budget code and title), or (iii) an external source. If the project has not already been fully costed for a funding application, please discuss the resources needed with the External Grants Coordinator, Finance Manager and the Director of Operations.

As a brief guide:

Staff - please indicate which staff members will be working on the project and the percentage of their time they will commit. If new staff are required please indicate the grade at which the appointment will be made and whether recruitment is internal(existing staff currently on another project) local(The Gambia) subregional(West Africa) or international. For new staff to be employed by MRC, full staff costs (including social security contributions, recruitment etc) may be obtained from the Personnel Manager, or from a spreadsheet operated by the External Grants Coordinator.

Consumables – these include all laboratory consumables, medicines and other clinical supplies, questionnaire production, computer consumables, specialist stationery and other supplies particular to the project. Freight costs should be included. The Purchasing Department and Laboratory Manager will advise on costs.

Access to existing equipment- Please specify e.g number of computers, and for example access to vehicles

Capital and minor equipment – any equipment that needs to be bought, replaced or repaired for the project. Freight and installation costs should be included. For

projects at the MRC Laboratories, the Laboratory Manager will advise about the availability of laboratory equipment.

Laboratory services- MRC laboratories has facilities for HLA typing, clinical microbiology (including TB) and routine haematology and biochemistry. Please indicate the number of samples to be processed in each area

Transport – if MRC transport is requested please indicate approximately how many kilometres of travel will be required. The Transport Manager will advise on the most cost-effective way of meeting the need given the resources available (vehicles, drivers etc). You should allow for local public transport costs for staff or study subjects , and night allowances for staff.

Space - indicate the requests for office space, freezer space(including liquid nitrogen storage) and laboratory space implied by the project and how it is suggested that these are met in discussion with the Scientific Administrator

Conference visits and Presentations- The Unit is particularly keen to support presentations by higher degree students and scientific officers. Please make the case here

Other – this may include, particularly for externally-funded projects, accommodation costs, clinical fees and other overhead charges, communication costs, training, meeting costs, library. Please discuss the availability of residential accommodation with the Housing Manager (short stays) or Director of Operations (longer term) and office space with the Scientific Administrator.

D2 Resource Request Spreadsheet(available in Excel on request)

TIMELINE
Please indicate the period of activity of the project from February 2006 –December 2007

D3 Sources of funds

If external funds have been or will be sought, please state the progress of the application(s).

Gates Malaria Partnership will provide £200K and the rest of funding will come from MRC laboratories.

Delivery of IPTc	**Dec-05**		
	nos. required	Year 1 Cost £	Year 2
Research Clinician (D3) West African post	1	10850	1188
Data Manager (D2)	0.5	3925	4318
Data Supervisor	1	2800	3080
Slide reader(B1)	4	7600	4180
Field Supervisors (B3)	2	4900	2695
Field workers (B1/B2)	6	13200	7260
Field workers (B1/B2) for 6 months	8	8800	
Data entry Clerks (B3)	4	9800	5390
Nurses (SEN) B3	5	16000	
Drivers(B2)	2	4400	2420
Allowance for IPTc dispenser	14	1000	
Allowance for IPTc officer	4	2400	
Sub-total		**85675**	**30531**
Staff related expenses			
Recruitment displaced West African		5000	
Recruitment cost local		500	
Medical care staff (3% of total salary)		2570	
Accommodation/person		4500	
Training staff (3% of total salary)		2570	
Air passage displaced West African		3000	
Shipment personal effects		3000	
Working visit/ conferences		9000	
Residential Permits per annum per family		500	
School fees Group D & E Staff		2000	
sun total		**32640**	

Transport			
Fuel for 2 landrover		7000	5000
Hiring of 14 motorbikes	14	8167	3500
Local travel & subsistence		3000	
Transport fares study subjects		3000	
Subtotal		**21167**	8500
Supplies			
Laboratory reagents & consumables		8000	
Stationery & computing consumables		3000	
Rapid antigen test		6000	
Permethrin for study subjects		3000	
subtotal		**20000**	
Clinical & costs associated study subjects			
Medical care for study subjects		6000	
Communications		2000	
Equipment			
6 Hemocue machine and cuvettes		5000	
2 microscopes		5000	
5PC plus 2 printers		6000	
Subtotal		**24000**	
Study drugs		12000	
Total			
**Cost effectiveness**			
Fieldworkers (2 x FT)	2	4400	
Health economist consultancy fee		8000	
International (3 x London/Gambia; 1 x Australia/Gambia		3000	
6 weeks @ £25 per day allowances OS staff		1050	
6* cassette recorders & tapes		500	
Supplies for FDG Participants		100	
Batteries		200	
Torches		100	
Mosquito Repellents		50	

Printing of Questionnaires	500
Writing Materials	400
Field Bags	100
Jackets/Uniforms	250
Mobile phones & calls	500
hiring 2 motorcycles	1167
**Subtotal**	**20317**
**Grand total**	£254830

Subject Information sheet

MRC Laboratories

Study: Comparison of two strategies for the delivery of IPTc in an area of seasonal malaria transmission

(Sponsored by Gates Malaria Partnership, London)

Subject/Patient Information Sheet

Seek consent from the parent/guardian of the child using the following explanation and in a language that he/she understands:

Malaria is an important cause of death and serious illness among Gambian children. However, the risk of malaria can be reduced by dugs and impregnated bed nets.

Recently, it has been shown that giving a full treatment dose of an anti-malarial drug at specific times, regardless of the presence or absence of malaria germs in the body can reduce the risk of malaria and anaemia in infants. MRC and partners are carrying out a study to find the best way in which this type of preventive treatment can be applied to protect infants and older children.

We want to find 12000-14000 children aged 3 months to 5 years to take part in this study. The study has been approved by the Joint Gambia Government/MRC Ethical Committee.

If you agree that your child can join the study, he/she will receive monthly treatment with two different types of antimalarial drugs (Fansidar and amodiaquine) during September, October and November from your local maternal and child health (MCH) clinic or your village. In addition, 1014 children will be randomly selected to take part in cross-sectional surveys at the end of the malaria transmission season. The duration of the trial is about one year.

If your child is selected to take part in the December cross-sectional survey, we will ask him/her for one finger-prick blood sample. This test will not do your child any harm but may cause some pain at the site where blood is taken. In addition, we will take a finger-prick sample from him/her whenever he/she develops fever and /or symptoms compatible with malaria.

If your child is sick at any time during the trial, he/she will receive free treatment at the health centre. Any costs you might have had to pay for transportation to the health centre will be reimbursed to you.

Your child need only join this trial if you want to. You can ask as many questions as you like and your child can leave the trial at any time without you giving any reasons.

If the study doctor thinks that your child should no longer take part in the trial for health or other reasons, he can end his/her participation.

Study: Comparison of two strategies for the delivery of IPTc in an area of seasonal malaria transmission

CONSENT FORM

Child's name…………………………………… Subject study number ____________

The information sheet has been read to me/I have read and understood the information sheet and I have had a chance to ask questions about the study.

I understand that my child will be asked to take treatment for malaria once every month for 3 months

I understand that if my child gets sick at any time during the trial, he/she can go to the local health centre to receive treatment from the health centre and MRC staff there. Information about the child will remain confidential and will be used only for the purposes of the trial.

I understand that my child will be asked to give finger-prick blood samples at the end of the study.

I understand that my child does not have to take part in the trial, and that he/she can leave the trial at any time, this would not affect the health care he/she or the immediate family receive while the trial is going on.

Signature or thumbprint of Parent or guardian of the

child… …………………………...

I have read the above to_____________________(name of parent or guardian of the

child) in a language he/she understands. I believe he/she has agreed that his/her child can take part in this study.

Signature of Field worker supervisor (or designate): _______________________

Date: __/____/____

Name in capitals: ________________________________
